# Major advances in targeted protein degradation: PROTACs, LYTACs, and MADTACs

**DOI:** 10.1016/j.jbc.2021.100647

**Published:** 2021-04-09

**Authors:** Shanique B. Alabi, Craig M. Crews

**Affiliations:** 1 Department of Pharmacology, Yale University, New Haven, Connecticut, USA; 2 Molecular, Cellular, and Developmental Biology, Yale University, New Haven, Connecticut, USA; 3 Department of Chemistry, Yale University, New Haven, Connecticut, USA

**Keywords:** PROTACs, molecular glues, LYTACs, AUTACs, chemical biology, protein degradation, ubiquitination, drug action, lysosome, ASGPR, asialoglycoprotein receptor, CDK, cyclin-dependent kinase, cIAP, cellular inhibitor of apoptosis protein, CRL, Cullin RING ligase, DNP, dinitrophenol, EGFR, epidermal growth factor receptor, FAK, focal adhesion kinase, FBnG, fluorobenzylguanine, FLT-3, FMS-Like tyrosine kinase 3, HECT, homologous to E6AP C-terminus, IGF-1, insulin-like growth factor 1, LYTAC, lysosomal-targeting chimera, M6P, mannose 6-phosphate, POI, protein of interest, PROTAC, proteolysis-targeting chimera, RING, really interesting new gene, RBR, RING-in-between-RING, TPD, targeted protein degradation, UPS, ubiquitin-proteasome system, VHL, Von Hippel–Lindau

## Abstract

Of late, targeted protein degradation (TPD) has surfaced as a novel and innovative chemical tool and therapeutic modality. By co-opting protein degradation pathways, TPD facilitates complete removal of the protein molecules from within or outside the cell. While the pioneering Proteolysis-Targeting Chimera (PROTAC) technology and molecular glues hijack the ubiquitin-proteasome system, newer modalities co-opt autophagy or the endo-lysosomal pathway. Using this mechanism, TPD is posited to largely expand the druggable space far beyond small-molecule inhibitors. In this review, we discuss the major advances in TPD, highlight our current understanding, and explore outstanding questions in the field.

Cells have evolved a precise orchestration of protein expression and degradation. While some protein half-lives are minutes long, others are maintained for days (1, 2). Furthermore, to ensure proper function, there are quality control systems that survey and remove misfolded proteins (3). This balance of protein health, known as proteostasis, is maintained by an extensive network (4). Ribosomes and chaperones are important for the production, folding, and maintenance of proteins, and the ubiquitin-proteasome system (UPS), endolysosomal and macroautophagic pathways work to break down proteins when necessary.

Scientists in a myriad of fields have performed formative work to illuminate key proteins and pathways in proteostasis (5). Over the past 20 years, chemical biologists have capitalized on this knowledge base to revolutionize the future of therapeutics. Using a bifunctional approach where a small molecule induces an interaction between a protein of interest (POI) and a component of the proteostasis machinery, chemical biologists can now control protein degradation. While early examples hijack the UPS to induce proteasomal degradation, more recent studies have shown that the endolysosomal and macroautophagic degradation pathways can similarly be co-opted for targeted protein degradation (TPD).

In this review, we discuss the different forms of TPD, highlighting the major advances in proteasomal-, lysosomal-, and autophagy-targeting technologies. We explain the current understanding of the mechanism of each technology and discuss the outstanding questions in each approach. Lastly, we discuss the future of TPD and its potential as a therapeutic strategy.

## Hijacking of the UPS: PROTACs

**Figure 1:**
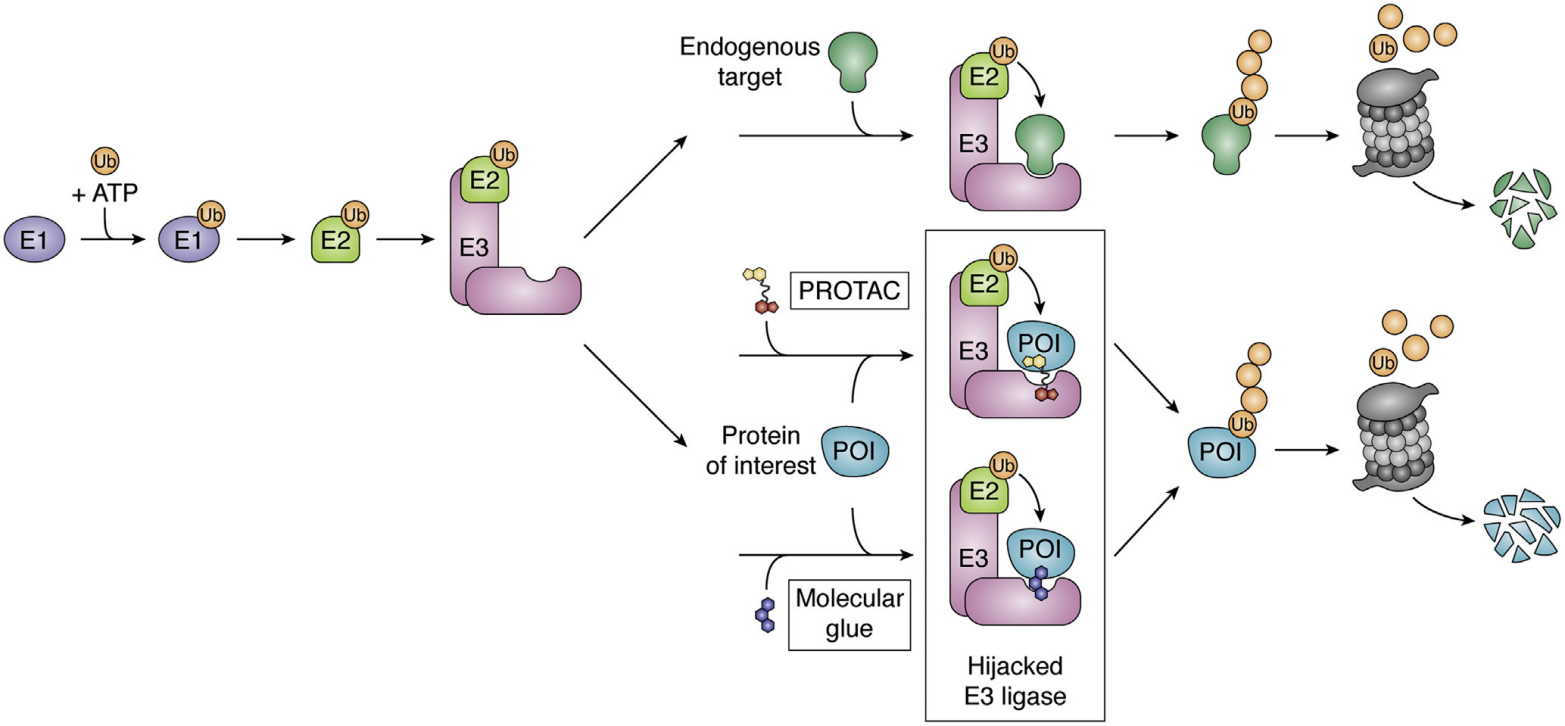
**Hijacking the ubiquitin-proteasome system (UPS).** In the first step of degradation *via* the UPS, the E1 activating enzyme forms a thioester bond with ubiquitin in a reaction powered by ATP. Through thioesterification, the ubiquitin molecule is transferred to an E2-conjugating enzyme. Lastly, by simultaneous engagement of both an E2 and the target protein, the RING E3 ligase coordinates the ubiquitination of lysines on the target POI. Polyubiquitinated POIs are then shuttled to the proteasome for degradation. PROTACs and molecular glues hijack the E3 ligase and recruit neosubstrates for ubiquitination and subsequent degradation.

The UPS is an essential pathway in the cell that processes the degradation of damaged or misfolded proteins (6, 7, 8). Maintenance of the UPS is critical for cellular function, and its dysregulation can lead to disease (7). To induce degradation, critical enzymes within the UPS posttranslationally tag proteins with ubiquitin, an 8.6 kDa stable protein with a beta-grasp fold. The cascade is initiated by a ubiquitin-activating enzyme (E1) that, through an ATP-dependent reaction, forms a thioester bond with ubiquitin. The ubiquitin molecule is then shuttled from the E1 to an E2 conjugation enzyme *via* trans-thioesterification. Lastly, the E3 adaptor protein facilitates transfer of ubiquitin from the E2 to a lysine residue side chain on the substrate protein (9). While Really Interesting New Gene (RING) E3 ligases coordinate ubiquitin transfer directly from the E2 enzyme to the target protein through the simultaneous binding of both, RING-in-Between-RING (RBR) and Homologous to E6AP C-Terminus (HECT) E3 ligases form a transient covalent interaction with the ubiquitin molecule and catalyze the final ubiquitin transfer to the target protein (10). Ubiquitin itself contains seven lysine residues. If these are the sites of further ubiquitination, this cascade leads to the formation of polyubiquitin chains on the target protein. The structural variation of these chains (*i.e.,* which lysine residue interconnects the multiple ubiquitin molecules) creates a “ubiquitin code” that signals for distinct cellular fates of the target (Fig. 1). For example, K48 chains primarily signal for proteasomal degradation, and K63 chains have been shown to promote lysosomal degradation (9, 11).

In 2001, our laboratory, in collaboration with the Deshaies laboratory, showed that the UPS can be hijacked to control protein degradation (12). In a proof-of-concept study, our group laid out the framework of PROTACs, PROteolysis-TArgeting ChimeraS (Fig. 1). The first PROTAC, (Protac-1) was composed of a β-TRCP E3 recruiting peptide linked to ovalicin, a small-molecule inhibitor that covalently binds the enzyme MetAP-2. Using lysates, we showed that Protac-1 can target and induce ubiquitination of MetAP-2. Encouragingly, Protac-1 also successfully induced degradation of MetAP2 in *Xenopus* egg extracts (12). In follow-up studies, we utilized microinjection of peptidic PROTACs (13) and a poly-*D*-arginine sequence to induce degradation in intact cells (14). While these early PROTACs were far from drug-like, they established the technology and highlighted that E3 ligases are susceptible to reprogramming and can mediate *de novo* degradation of substrates they do not naturally degrade (neosubstrates).

However, the finding that propelled the field forward was the development of an all-small-molecule PROTAC. This PROTAC utilized nutlin, a small molecule that binds the E3 ligase MDM2 to induce degradation of the androgen receptor (15). To date, PROTACs have been used to target a plethora of proteins in various compartments of the cell (16, 17, 18, 19). In addition, PROTACs have been shown to successfully degrade major disease-causing proteins that have traditionally eluded the pharmaceutical industry such as tau (20, 21) and KRAS (22, 23, 24). Furthermore, the establishment of Halo-PROTACs (25) and dTAG (26, 27) systems, which allow targeted degradation of HaloTag7 and FKBP fusion proteins respectively, has allowed for degradation of proteins without established ligands. The breadth of proteins targeted by PROTACs has been extensively reviewed elsewhere and thus will not be the focus of this article (28, 29, 30). Here, we collectively review the major advances in PROTAC technology through case studies and highlight the important principles that guide successful PROTAC development.

### Identification of E3 ligase recruiting elements

There are ∼600 predicted E3 ligases; however, the function of many is yet to be fully characterized (31). Moreover, the substrate-binding interface for E3 ligases is often shallow and large, making finding a druggable pocket a difficult feat (32). In fact, E3 ligases were considered “undruggable” and traditional inhibitors were only reported within the last decade (33, 34, 35).

To develop PROTACs, one can take advantage of the plethora of established small molecules designed to bind disease-causing proteins. Conversely, prior to TPD, there was minimal incentive to develop E3 ligase ligands that did not impair E3 ligase function. Thus, E3 ligase recruiting elements incorporated into PROTACs are often repurposed inhibitors or identified serendipitously. The nutlin group of compounds were discovered through a high-throughput screen to inhibit MDM2-p53 interactions (36). By binding the substrate-binding cleft of MDM2 nutlin prevents p53 degradation. As nutlin does not inhibit MDM2, it was a great candidate E3 ligase ligand for the first all-small-molecule PROTAC. Following the report on nutlin-based PROTACs, bestatin esters—small-molecule ligands of the cellular inhibitor of apoptosis protein (cIAP) E3 ligase—were also shown to be amenable for use in TPD (37). Bestatin was initially isolated from *Streptomyces olivoreticulithe* as an inhibitor of aminopeptidases and later found to bind IAP proteins. The IAP family are antiapoptotic proteins that inhibit caspase activity (38) and also serve as RING E3 ligases with neosubstrate degradation capabilities (39, 40, 41, 42, 43, 44, 45, 46). The Hashimoto group named this subset of PROTACs, SNIPERs: Small and Nongenetic Inhibitor of apoptosis protein (IAP)-dependent Protein Erasers (39). Since then, other IAP ligands with more drug-like properties have been developed, such as MV1 (47) and LCL161 (48, 49, 50, 51). It is noteworthy that IAP E3 ligase recruiting molecules induce IAP autoubiquitination and degradation, thus PROTACs that incorporate SNIPERs cause a self-limiting decrease in the IAP E3 ligase levels. However, IAP is a known oncogenic target, and therefore the dual targeting may offer added advantages in some scenarios (52).

In a direct effort to develop E3 ligase ligands, our lab conducted a structure-based drug design effort to design Von Hippel–Lindau (VHL)-binding small molecules (53, 54). VHL is a well-established E3 ligase substrate receptor, which tightly binds hydroxylated HIF-1α. With HIF-1α-based peptidic PROTACs, we previously determined that VHL was amenable to TPD (55). Thus, through rational design, we established a small molecule that contains functional groups that recapitulate the important VHL-binding interactions of the HIF-1α sequence. Crystal structures of the small molecule bound to VHL provided insight into suitable attachment points to create PROTACs (56). Indeed, several studies have now shown that this ligand can be incorporated to produce successful PROTACs (17, 24, 25, 57, 58, 59, 60, 61, 62, 63). In fact, the VHL ligand is one of the most used E3 ligase recruiting elements in the PROTAC field to date.

The discovery of thalidomide's mechanism of action as a cereblon (CRBN) ligand in 2010 suggested a second E3 ligase recruiting moiety for common use (64) (see Hijacking the UPS: molecular glues section for further elaboration). Indeed, thalidomide and its derivatives (the “phthalimides” or “IMiDs”) have since been used in PROTACs to induce the degradation of numerous target proteins (59, 64, 65, 66, 67, 68). Interestingly, as compared with VHL, the promiscuous CRBN has been shown to be more accepting of neosubstrate degradation thus far (69). However, this promiscuity is a bit of a double-edged sword in that PROTACs incorporating phthalimides are not as selective and can induce degradation of nonintended neosubtrates such as G1 to S phase transition protein 1, a protein identified as a common off target of IMiD-based degraders (29, 70).

MDM2, CRBN, VHL, and IAP are the most frequently recruited E3 ligases in PROTAC development (29). However, we have shown that other E3 ligases including HECT and RBR family members can be hijacked to induce degradation of neosubstrates (71).Thus, there is ample space for expanding the toolbox of ligands to target different E3s. Recently, much effort has been placed in expanding the E3 ligase repertoire. Many of these studies have adopted chemoproteomic approaches to identify covalent E3-ligase binders (29).

To this end, the Nomura group used activity-based protein profiling (ABBP) to identify CCW 16, a covalent binder to the E3 ligase RNF4 (72). By attaching CCW-16 to JQ1, a Bromodomain and Extraterminal (BET) family inhibitor, they show successfully induced degradation of BRD4 enabled by RNF4-mediated ubiquitination. Also, covalent fragments that label a cysteine of the E3 ligase DCAF16 using ABBP have been identified by the Cravatt group (73). This E3 ligase is localized to the nucleus and thus induces nuclear selective degradation of BRD4 when linked to JQ1, exemplifying the utility of hijacking E3 ligases that can furnish subcellular-specific degradation. Both groups found that the level of E3 ligase labeling was low (∼30% for RNF4 and ∼10–40% for DCAF16). Despite this low level of engagement, the PROTACs induced substantial degradation of their targeted proteins. Following the early success seen with covalent PROTACs, the Nomura group incorporated bardoxolone, a reversible covalent binder of the E3 KEAP1, in a JQ1-bardoxolone PROTAC that afforded nanomolar BRD4 degradation (74). Nimbolide, a natural product extracted from neem leaves, which acts as a RNF114 E3 ligase recruiting ligand, has also been used to induce degradation of BRD4 and BCR-Abl when linked to JQ1 and dasatinib, respectively (75, 76). Although the newly identified E3 binders may require optimization for specificity, they illustrate that E3 ligases are generally susceptible to hijacking for TPD. Whether these covalent PROTACs can degrade their targets in a substoichiometric fashion has yet to be formally established, although their surprising potency strongly hints at a catalytic mechanism (Fig. 1). Nonetheless, through chemoproteomics the number of E3 ligases one can recruit has increased substantially in a few years.

Through expansion of the recruitable E3 ligases, the specificity of effect that PROTACs achieve may be greatly increased. For instance, one can envision selectively targeting transcription factors in the nucleus at its site of action leading to more desirable pharmacological effects. Furthermore, using disease-specific E3 ligases, one could target essential proteins that may be toxic to degrade systemically. Lastly, as some E3 ligases have been shown to form K63 ubiquitin chains, it may be possible to recruit these E3 ligases and promote lysosomal degradation as opposed to proteasomal degradation.

### Catalytic mechanism of action

Over the past 20 years, significant efforts have been made to elucidate the PROTAC mechanism of action and develop principles and guidelines for TPD. When PROTACs where first established, they were posited to have the ability to function catalytically, *i.e.*, one PROTAC molecule in theory should be able to coordinate degradation of more than one equivalent of protein molecule (77). This is in opposition to small-molecule inhibitors, which operate in a stochiometric fashion.

To establish this, we studied degradation of RIPK2, a kinase involved in innate immunity and associated with sarcoidosis (78). In an *in vitro* ubiquitination assay with radiolabeled RIPK2, we were able to quantify the moles of PROTAC-induced RIPK2 ubiquitination and compare them to the moles of PROTAC in each reaction (79). Indeed, the experiment showed that each PROTAC was able to mediate the ubiquitination of 2 or more RIPK protein molecules, thus establishing that PROTACs act *via* a multiple turnover mechanism. Since then, studies have shown that a single PROTAC treatment can induce degradation over the course of several days, offering further supporting evidence that PROTACs induce degradation substoichiometrically.

However, due to their large size, the catalytic activity of PROTACs is at times dampened by poor cell permeability and possibly decreased binding affinity (due to linker addition) (80). Despite these limitations, several studies have shown very potent, even picomolar-range PROTAC-mediated degradation of targets (79, 81).

### Linkerology and ternary complex formation

Simplistically, PROTACs must contain a POI-binding ligand, a linker, and an E3 ligase recruiting element. In practice, however, PROTAC development is far from simple. To successfully induce degradation, there are several important factors that must be considered.

**Figure 2:**
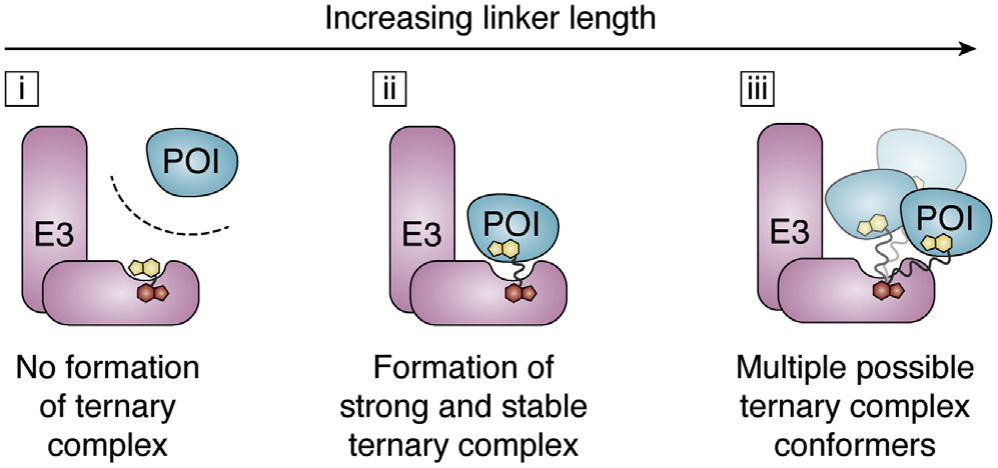
**Linkerology and ternary complex formation.** The linker length plays an important role in PROTAC-induced degradation. (i) Short linkers that do not allow each target protein to engage its respective ligand will not allow ternary complex formation and thus will fail to induce degradation. (ii) The ideal linker length(s) will allow for strong and stable ternary complexes to form with positive cooperativity. (iii) As linker length increases, due to increased flexibility, the number of possible ternary complex conformers increases. This increased flexibility is tolerated differently by each E3:POI pair. Thus, the“ideal linker” will vary for each neosubstate and E3 pair.

A very important parameter in PROTAC development is the linker length and composition (25). In addition to influencing pharmacokinetic properties, the linker plays an important role in influencing the pharmacodynamics of a degrader. A simple study that highlights linker significance was our quest to create HaloTag-based PROTACs (“HaloPROTACs”). HaloTag is a bacterial dehalogenase that covalently binds chloroalkanes. To create our HaloTag-degrading PROTACs, we linked a chloroalkane to a VHL-recruiting ligand with polyethylene glycol (PEG) linkers of increasing length. Our studies showed that linkers that were too short (less than three PEG units) induced minimal degradation of HaloTag: with a maximal degradation (D_MAX_) ≤20%. However, as the linker length increased to three PEG units (HaloPROTAC3), the HaloPROTAC induced efficient degradation, with a D_MAX_ >95% and a DC_50_ (concentration of half maximal degradation) = 19 ± 1 nM. HaloPROTACs with longer linker lengths (greater than three PEG units) achieved a slightly lower D_MAX_ (∼80%), and at higher concentrations, a reversal of target degradation was observed. This is due to the “hook effect,” where at high concentrations binary species (E3:PROTAC or POI:PROTAC) rather than ternary complexes (E3:PROTAC:POI) dominate. Conversely, the hook effect was not observed with the HaloPROTAC3 to 10 μM. This study with haloPROTACs showed that, despite being constructed of two very well-established recruiting ligands, small changes in linker length can drastically alter the degradation efficiency. Furthermore, a nonideal linker (too short or long) can strongly affect the window of concentrations at which a PROTAC is effective. A second lesson from this study is that for efficient and potent degradation, a PROTAC must have a long enough linker to allow for ternary complex formation, but short enough to discourage “non-ideal” ternary complexes, which decrease the window of maximal effect (Fig. 2).

Similarly, in studying Bruton's tyrosine kinase (BTK) degradation, Zorba *et al.* (67) found that while shorter linkers (PEG units <5) discouraged degradation, longer linkers were more successful. They posit that shorter linkers were too short for both warheads of their PROTAC to be accessible at the same time to the binding pockets of both proteins, discouraging ternary complex formation. However, at a critical linker length (PEG units ≥5), they observe successful degradation of BTK, with nine PEG units inducing the most potent degradation (DC_50_ = 5.9 ± 0.5 nM). To explore this relationship more concretely, they compared degradation efficiency to ternary complex formation. Using a TR-FRET ternary complex assay, they found the PROTACs with short linkers, which failed to induce degradation of BTK, also formed very poor ternary complexes. However, PROTACs with five PEGs and above did. We observed similar linker dependencies in studying TBK degradation (82). Thus, ternary complexes are a strong indicator of successful degradation.

A second important function of the linker is establishing binding cooperativity. In the PROTAC field, cooperativity is defined as the effect that protein association (ex. POI:PROTAC) has on the affinity of the PROTAC to the second protein (the E3 ligase). Mathematically it is defined as (α = K_D_^binary^/K_D_^ternary^) (83). Positive cooperativity occurs when the K_D_^ternary^ is smaller than the K_D_^binary^. This is often accomplished by the addition of stabilizing interactions between the POI and the E3 ligase. Conversely, negative cooperativity occurs when the K_D_^ternary^ is larger than the K_D_^binary^ due to destabilizing interactions between the POI and E3 ligase. Increased cooperativity, in theory, stabilizes the ternary complex (POI:PROTAC:E3 ligase) and can potentially decrease the magnitude of the hook effect (59).

To explore the role of cooperativity in ternary complex formation, the Ciulli group studied PROTAC-induced degradation of the BET protein family (BRD2, BRD3, BRD4) (83), a protein family extensively studied in the PROTAC field (27, 60, 73, 74, 81, 84, 85, 86, 87, 88, 89, 90, 91, 92, 93, 94, 95, 96, 97, 98, 99, 100, 101). Using SPR and ITC, they found that ternary complexes with positive cooperativity (BRD4^BR2^:PROTAC MZ1:VHL; α = 22) caused more efficient cellular degradation than complexes with negative cooperativity (BRD4^BR2^:PROTAC MP61:VHL; α = 0.2). High cooperativity is characterized by fast on rates, slow off rates, and therefore longer ternary complex half-lives. Similar results were observed by Riching *et al.* (102) using Nano-BRET.

Conversely, Zorba *et al.* found that the PROTAC that exhibited the highest cooperativity (α = 2.5) did not yield the most efficient degradation of BTK. Rather, the most potent and efficient degraders (PEG units ≥5) exhibited little to no cooperativity (α ∼ 1). They hypothesize that the positive interactions gained in their ternary complex may be offset by the entropic cost of their flexible linker. Thus, as linker lengths increase, the number of possible ternary conformers that can exist increase. This increase may have a minimal effect on some neosubstrate but can cause a decrease in degradation efficiency in others (Fig. 2, iii). As such, the ideal linker length varies for each target, and the ideal linker will need to be determined on a case-by-case basis. Furthermore, the linker composition and vector (the direction in which an E3 ligase is recruited to a protein) can play important roles in encouraging or discouraging ternary complex formation (103, 104). Thus, while cooperativity is highly correlative of enhanced degradation, it will not always predict the most efficient and potent PROTAC.

### Structural insights into cooperativity and linkerology

Structural biology is a vital component of drug discovery. In TPD, atomic resolution structures have been important in selecting the ideal vector for linker addition. However, to date there are few guiding principles for determining an ideal linker length.

To gain a better understanding of the molecular determinants that control ternary complexes, Gadd *et al.* (58) crystalized the first 2.7 Å PROTAC ternary complex structure of BRD4^BD2^:MZ1:VHL. The structure revealed that MZ1, a PROTAC with a ten-atom linker, was significantly folded to allow the two proteins to associate, though there were some positive interactions between the linker and both proteins. Indeed, they found that the surface complementarity of the two proteins played a significant role in the newly formed ternary complex. There existed an extensive interface of the two proteins of 688 Å^2^ facilitated by hydrophobic and charged zipper contacts. In fact, decreasing surface complementarity through point mutations on BRD4^BD2^ at the interface caused a decrease in ternary complex formation as estimated using TR-FRET and ITC. With insights from the ternary structure, they designed a next-generation BRD4 degrader, AT1, composed of a shorter linker, which induced selective BRD4 degradation while sparing BRD2 and BRD3. More recently, Testa *et al.* (99) designed macrocyclic PROTACs (macroPROTAC-1) with the goal of similarly stabilizing the ternary complex. A solved 3.7 Å structure showed macroPROTAC-1 occupied a near-identical BRD4^BD2^:VHL conformation (with a 681 Å^2^ interface) as compared with MZ1. As predicted, using this strategy they observe an increase in cooperativity with BRD4^BD2^, but decreased cooperativity with other members of the BET family leading to equally potent, but more selective degradation of BRD4.

In addition, Chung *et al.* (105) solved a 1.9 Å Bcl-xL:PROTAC-6:VHL ternary structure. Bcl-xL is a protein of the Bcl-2 family, important in regulating cellular apoptosis (106). Despite PROTAC 6 incorporating a general Bcl-2 family inhibitor, venetoclax (107), it induced selective degradation of Bcl-xL. Using SPR, they observed a stable yet uncooperative ternary complex (α= 0.76). Thus, PROTAC 6 induced potent degradation (DC_50_ = 4.8 nM; D_max_ =76%) of Bcl-xL, but also exhibited a hook effect at low concentrations (<300 nM).

The solved ternary complex structure shows that PROTAC 6 also adopts a folded configuration, allowing for an extensive 672 Å^2^ interface between Bcl-xL and VHL. Thus, these results suggest that there are likely several other possible ternary complexes that occur, and the negative cooperativity observed is not due to a lack of interaction between the two proteins, but an entropic penalty from the amalgamation of the possible ternary complexes. In fact, this might explain the strong hook effect observed. Similar dynamics are likely at play for PROTAC 9 in BTK degradation (Zorba *et al.*, described above) where no cooperativity was observed, but a HDX study showed stabilization of BTK in the presence of the PROTAC and VHL (67). As such, lack of cooperativity does not equate to a lack of an interaction between the POI:E3 ligase.

Overall, these studies highlight the immense potential and learning that can be achieved using atomic-resolution structures. For one, structures not only expose possible linker attachment points, but can also reveal the ideal linker length of a PROTAC. Furthermore, ternary complex structures strongly aid in optimizing cooperativity of a specific ternary complex. In doing so, one can so discourage recruitment of other proteins, allowing for increased selectivity.

### Computational modeling of TPD

In lieu of atomic-resolution structures, computational modeling has been used to make structural predictions and to rationalize PROTAC selectivity. In an early example, Smith *et al.* (103) used a short molecular dynamics (MD) simulation to help explain PROTAC selectivity of p38 isoform degradation. However, to investigate ternary complex modeling more broadly, Drummond *et al.* (108) sampled a few rational methods for assessing ternary complexes using the MOE modeling package. They found that independently exploring both PROTAC conformations and POI:E3 interactions (ideally with a structure in the ligand-bound conformation), and then combining these two models, yielded the most accurate crystal-like poses. Using this method, they successfully estimate existing ternary complex structures within a 10 Å RMSD and accurately predict the degradation ranking of WT *versus* mutant BRD4 proteins.

Conversely, Zaidman *et al.* (109) presented the PRosettaC model, which also utilizes consecutive steps to estimate ternary complex structures. Their method begins with ligand distance sampling using anchor atoms. Patchdock is then used to perform more constrained, local POI:E3 ligase docking, accounting for the distances obtained from ligand sampling. Next, RosettaDock is used for refinement and local docking of the complex. Lastly, PROTAC solutions compatible with the local dock are clustered and ranked to provide possible ternary complex solutions. Using this method, they recapitulated existing structures six out of ten times, with the near-native models ranking in their top three generated solutions. Furthermore, this method allowed for more accurate models than previously published computational methods in three out of five instances.

The Williams group has further refined their computational model using more constrained PROTAC conformations (110). As more atomic-resolution structures are solved, we anticipate that the accuracies of these models will be even further refined. These computational models will likely be an important tool in attempting to rationally design PROTACs. However, it is important that these models be utilized in conjunction with other experimental data. Beyond structural modeling, mathematical models are now being generated to understand the entire process of targeted protein degradation (111).

### Ubiquitination and degradation *via* the proteasome

The establishment of a thermodynamically stable ternary complex is important primarily because it allows for ubiquitin molecules to be transferred to lysine residues on the POI. Ubiquitination is established as a vital step for PROTAC-induced degradation (9, 59, 77, 79). Beyond this general observation, little has been explored and many outstanding questions remain to be answered, largely because there is still much to be understood about ubiquitination as a whole.

In an early study to investigate ubiquitination, we used mass spectrometry to investigate specific lysines on eGFP-HaloTag marked by different E3 ligases recruited by HaloPROTACs (71). Overall, we found that the specific residues as well as the total number of lysines marked with ubiquitin varied based on the recruited ligase. While VHL induced the ubiquitination of one lysine on eGFP, cereblon ubiquitinated three. The recent study by the Zhou group also investigates ubiquitination sites of BCL-xL PROTAC-mediated degradation (62). Using mass spectrometry and mutational studies, they identified a single lysine, K87, which is primarily marked with ubiquitin and allows for protein degradation. Mutations of the other lysines on the surface of Bcl-xL did not affect targeted degradation. Thus, a single lysine modification is sufficient to signal for degradation and can vary based on the E3 ligase recruited.

As the field of “ubiquitin-omics” expands, further investigation of lysine ubiquitination will need to be explored. Some general questions include: what is the threshold of ubiquitination that leads to degradation? What ubiquitin chain types (K48, K11, etc.) dominate? What is the ubiquitination zone for each E3 ligase? How does ubiquitination of neosubstrates differ from natural substrates? Can PROTAC-induced ubiquitination be used to signal outcomes other than degradation?

### Increased selectivity and decreased toxicity

The goal of creating selective drugs is to maximize on-target effects while minimizing general toxicity. Major challenges to the creation of selective drugs are protein families that contain common protein folds and high active site structural conservation. For instance, while kinases are involved in several disease mechanisms and kinase inhibitors have been developed and optimized for decades, generally kinase inhibitors are notoriously unselective. One such kinase inhibitor is foretinib, which at 10 μM binds 133 kinases. However, conjugation of this inhibitor to E3 ligase ligands yielded a VHL-recruiting PROTAC that induced significant degradation of only nine proteins and a CRBN-recruiting PROTAC that induced degradation of only 14 targets (59).

In this example, selectivity is built in at least two ways. Firstly, out of the 133 kinases, only 52 kinases retain binding to the VHL-recruiting PROTAC and 62 kinases to the CRBN-recruiting PROTAC. Thus, simply adding a linker and an E3 ligase binder decreases binding to more than half of the kinases normally bound by foretinib. Secondly, additional selectivity is achieved through PROTAC-induced ternary complex formation. For instance, although the SLK kinase engages the VHL-recruiting PROTAC, it fails to form a ternary complex and thus is not degraded. This result is consistent with the Zorba *et al*. study where ternary complex was not completely indicative of degradation. Hence, through differences in binding and the additional requirement of forming ternary complexes, PROTACs can achieve immense selectivity even with promiscuous binders/warheads (of the POI).

Somewhat surprisingly, this study using foretinib-based PROTACs also demonstrates that while ternary complexes are necessary to successfully induce degradation, they are not always sufficient. For example, c-Abl forms a stable ternary complex with, but is very weakly degraded by, the VHL-recruiting PROTAC. The mechanism that accounts for this result is not fully understood. However, recent examples have shown that in some cases where *in vitro* experiments show successful ternary complex formation, in cell ternary complex assays do not. This was observed by Khan *et al.* (62) where using an AlphaLISA assay to evaluate their PROTAC, they observed *in vitro* ternary complex formation with BCL-X_L_ and BCL-2 (the latter being a protein that is not successfully degraded by its PROTAC) and VHL. Conversely, in cells, they did not observe BCL-2 ternary complex formation using a nanoBRET assay. A similar phenomenon was observed by our group, as well as the Sicheri group, while studying PROTACs that induce selective mutant BRAF degradation but spare WT BRAF (112, 113). We found that *in vitro* studies showed PROTAC-dependent ternary complex formation with WT BRAF and VHL, while conversely *in cellulo* assays (nanoBRET) did not. The exact reason for these results remains unclear. However, one can speculate that ternary complex formation may be further complicated in cells due to more intricate protein conformation, posttranslational modifications, or protein complexes. Beyond this, one can also speculate that a lack of accessible lysines for ubiquitination may also factor into PROTAC selectivity. Thus, the additional steps required for successful PROTAC function as compared with protein inhibition, although cumbersome, can allow for several degrees of selectivity to be achieved.

Another proposed utility of PROTACs is that it can revive small-molecule campaigns where inhibitors were deemed too toxic or to have limited function. One such “possibly revived” drug class are the dual BCL-2/BCL-X_L_ inhibitors. The BCL-2 family of proteins (BCL-2, BCL-X_L_, and MCL-1) are antiapoptotic proteins overexpressed in cancers. While one BCL-2 selective inhibitor (venetoclax) is approved, BCL-2, BCL-X_L_ dual inhibitors such as ABT263 are limited due to on-target inhibition of BCL-X_L_ in platelets that causes thrombocytopenia.

Using ABT263-based, VHL-recruiting PROTACs, Khan *et al.* (62) show that BCL-X_L_ degradation is induced in T-cell acute lymphoblastic leukemia (T-ALL) tumor models but is spared in platelets. Their work showed that the cell-type-specific BCL-X_L_ degradation they observed is due to the low levels of VHL expression in platelets, which results in nominal PROTAC activity there. In additional studies, they similarly showed that CRBN levels are also decreased in platelets, and thus phthalimide-based PROTACs also induced platelet sparing degradation (114). In addition, BCL-2 is also spared from degradation in all cell types (62, 63, 115, 116). Thus, using a degradation-based strategy, a target in cancer—BCL-X_L_—that could not easily be safely addressed by simply inhibition is now therapeutically viable.

### Enhanced and differential biology

While small molecules often only inhibit enzymatic activity, PROTACs completely remove the POI. This allows targeting of both enzymatic and nonenzymatic roles of proteins. Studies have shown that complete removal of proteins can cause a more pronounced effect than inhibition. For example, degradation of Epidermal Growth Factor Receptor (EGFR) and FMS-Like Tyrosine kinase 3 (FLT-3) caused more sustained inhibition of their respective signaling pathways leading to decreased cell growth (17, 117). Similarly, BRD4 degradation causes more suppression of c-MYC, its downstream target, levels than BRD4 inhibition (81). As more studies explore transcriptome level details on cells treated with inhibitors *versus* PROTACs, it is likely that unrealized functions of proteins will emerge.

Over the past few years, several examples of “differential biology” have been observed wherein targeting a protein for degradation resulted in a biological effect distinct from the one observed from its inhibition. For example, while inhibition of Focal Adhesion Kinase (FAK) with defactinib inhibits cell division, a defactinib-based PROTAC halted both cell proliferation and cell motility (118). Furthermore, a more recent study found that while inhibition of Aurora A with alisertib caused G2/M arrest, degradation with an alisertib-IMiD iMID-based PROTAC rather induced S phase arrest. The latter led to an increase in apoptosis. Interestingly, however, the study found that CRBN interacts with Aurora A kinase even in the absence of the PROTAC (2). However, whether CRBN is a natural E3 of Aurora A kinase is unknown.

### Overcoming resistance from small-molecule inhibitors

Cellular adaptation to targeted therapies has long been an issue in cancer therapeutics (119). Despite years of work and funds invested to develop safe and effective therapies, resistance is developed to many drugs used in the clinic. In oncology, resistance has been shown to occur in as little as a few months after treatment. Furthermore, resistance to drug treatment can be intrinsic due to innate chraracteristics, thus the drug becomes ineffective even prior to the beginning of the treatment. Mechanisms of resistance include increased drug efflux, inhibition of apoptotic or autophagy pathways, changes in drug metabolism and compartmentalization, and changes in drug target or drug target levels.

Targeted protein degradation has overcome some resistance mechanisms that cause changes to the drug target. For instance, enzalutamide is a successful FDA-approved drug that targets castration-resistant prostate cancer *via* androgen receptor antagonism; however, inevitably resistance occurs (120). Mechanisms of resistance include androgen receptor point mutations that alter the effectiveness of enzalutamide. In fact, point mutations such as F876L and T877A turn enzalutamide into an agonist (121, 122). However, using an enzalutamide-based PROTAC, ARCC-4, we showed degradation of proteins presenting several of these mutations observed in clinic (80). A second mechanism of action observed in CRPC is androgen production in tumor cells, which outcompetes binding of the drug to the androgen receptor. While 1 nM of R1881 (an androgen mimetic) overcomes the effect of enzalutamide due to decreased drug target engagement, ARCC-4 still induces degradation of the androgen receptor to block prostate cancer cell growth even in the presence of 10 nM R1881. As transient interaction with the target is sufficient to induce its destruction, PROTACs can have a therapeutic effect in conditions where small molecules that depend on sustained target interaction are ineffective.

In a different example, we and others showed that an ibrutinib-based PROTAC can induce degradation of an ibrutinib-resistant C48S BTK mutant (18, 86). Ibrutinib is a covalent inhibitor that reacts with Cysteine 48 on BTK. Thus, the C48S mutation abolishes covalent binding: BTK C48S is still capable of binding ibrutinib, albeit noncovalently, rendering the inhibitor ineffective as a therapy. However, by incorporating ibrutinib into a cereblon-recruiting PROTAC and using a degradation-based strategy, we can successfully target mutant BTK-driven cancer. More recently, we have shown that PROTACs can also be useful in conditions where the mutant is intrinsically resistant to a parent inhibitor (113). Using vemurafenib-based PROTACs, we show not only induced degradation of BRAF V600E, the FDA-approved target of vemurafenib, but also degradation of additional missense BRAF mutations that are intrinsically resistant to the vemurafenib warhead. This notably includes mutants that have little to no kinase activity (Class 3 BRAF mutations).

These studies highlight a large benefit of PROTACs. Small molecules must bind and stay bound to their targets to halt enzymatic function of the protein. Thus, any modification that hinders that occupancy dampens the effect of the small molecule. However, PROTACs work *via* an “event-driven” pharmacological mechanism whereby clinical benefit is not proportional to occupancy, but rather to the ability to ubiquitinate. Therefore, if the PROTAC molecule can bind to its target even only transiently, it has the potential to be effective as a therapeutic. However, in the presence of target mutations that completely abrogate binding to the warhead, the PROTAC mechanism will be rendered ineffective.

### Resistance to PROTACs

Despite their potential to overcome inhibitor resistance mechanisms, as PROTACs enter the clinic it is likely that patients will also develop resistance to this new modality. Therefore, it is important that the field understands the ways tumors can and will overcome the technology.

Recognizing the importance of this, Mayor-Ruiz *et al.* (123) performed a positive selection CRISPR/Cas9 screen for five TPD drugs that utilize different Cullin RING ligases (CRL), namely DCAF, VHL, and CRBN. Their goal was to delineate effectors that are required for successful TPD and outline putative resistance mechanisms. They identified that TPD resistance is driven primarily by changes to the CRL that is recruited and not by the target protein that is degraded. Furthermore, CRL-based resistance mechanisms are context-specific. For instance, CAND1/CSN, proteins important in regulating RING ligase plasticity, are a major determinant of CRL4-based degraders (DCAF15 and CRBN), but not those that act *via* CRL2 (VHL). In fact, loss of these regulatory proteins led to CRBN autodegradation. VHL resistance was mostly mediated by loss of UBE2M, an NEDD8 E1 conjugation enzyme.

In a more direct approach, Zhang *et al.* (61) established resistance to both VHL-based (ARV-771) and CRBN-based (ARV-825) BET PROTACs by treating cancer cells with increasing concentrations of either drug for a 4-month period. While not all cell lines successfully became resistant, they found that OVCAR8 cells separately formed resistance to either PROTAC. Interestingly, the VHL-based PROTAC maintained activity in the CRBN-recruiting PROTAC-resistant cell lines, and the CRBN-recruiting PROTACs maintained activity against the cells that were resistant to the VHL-recruiting PROTAC. In studying the mechanism of resistance using whole exome sequencing, they also found that resistance primarily involved alterations to the corresponding E3 ligase machinery rather than alterations on BET. In ARV-825 resistant cells, this was mediated by downregulation of CRBN itself. This result is in line with resistance seen in myeloma patients treated with IMiD drugs (124). Interestingly, in ARV-771-resistant cells, resistance was rather mediated by downregulation of Cullin2, but not VHL. In a similar study using the AML cell line, MV4-11, Ottis *et al.* (125) also raised resistance to CRBN- and VHL-engaging PROTACs and found near-identical results.

Thus, these studies identify the possible biomarkers one can use for assessing resistance to TPD. Furthermore, they highlight the importance of identifying additional E3 ligase recruiting elements to overcome PROTAC resistance.

### Additional control of protein degradation

To gain better control of when and where PROTACs induce degradation, recent studies have explored utilizing additional strategies to regulate TPD. Early examples explored light-induced degradation *via* PROTACs. In these systems—Photo-PROTACs (126), PHOTACs (127), and azo-PROTACs (126)—a photo-switchable linker is used to control PROTAC conformation. In the absence of light, the degrader adopts an inactive conformation, which is not amenable to ternary complex formation. However, upon irradiation at the correct wavelength, the linker conformation is altered, allowing degradation to occur. Additional studies such as opto-PROTACs (128) and pc-PROTACs (92) have utilized photo-labile groups where irradiation at a specific wavelength uncages the prodrug-PROTAC and allows for degradation. While this technology is in its infancy, it may allow for localized degradation within solid malignancies (129).

Furthermore, PROTACs have been coupled to antibodies to facilitate tissue-specific degradation. The Tate group demonstrated this by coupling a BRD4-targeting PROTAC to trastuzumab, a HER2-targeting antibody (85). Using this method, they show internalization of the PROTAC only in HER2 positive cells and thus achieve target degradation in those cells selectively. Antibodies have also been utilized to degrade endogenous proteins with technologies such as “Trim-Away” (130). More recently, nanobodies have also been coupled to RING proteins to achieve highly selective degradation of target proteins (131). Clinically, these additional controlled mechanisms on PROTACs may allow for localized degradation.

## Hijacking the UPS: molecular glues

Molecular glues are the second arm of targeted protein degradation *via* the proteasome. These degraders are small molecules that bind an E3 ligase, thus modifying its substrate recognition site allowing it to bind and degrade a neosubstrate. While PROTACs are capable of binding both the POI and E3 ligase independently, molecular glues engage the E3 ligase allowing that complex to recruit its target (Fig. 1). Thus, the neosubstrate does not require a ligandable pocket to be recruited for induced degradation. Furthermore, as the neosubstrate does not bind the molecular glue in the absence of the E3 ligase, molecular glues often exhibit positive cooperativity (α ≥ 1). The term molecular glue was first coined by the Zheng group while studying Auxin, a plant hormone that binds the F-box protein transport inhibitor response 1 (TIR1), and allows it to catalyze the degradation of Aux/IAA transcription repressors (AID) (132). Consequently, auxin is now used as a tool compound to study the degradation of proteins. However, as TIR1 and AID are not conserved in mammalian cells, they must be artificially introduced (133).

While the PROTAC field was launched with an intentional effort to design protein degraders, molecular glues were identified serendipitously. In fact, the first molecular glue drug, thalidomide, was approved in patients without knowledge of its ability to induce degradation. Thalidomide was first introduced in the 1950s and 1960s as an antinausea drug for pregnant women (134). Tragically, the drug was found to cause severe birth defects and was pulled from the market, revolutionizing the way drugs are tested and approved in the United States (135, 136). Decades later, thalidomide was revived again for several indications including leprosy, autoimmune diseases such as lupus, and multiple myeloma, but still without full knowledge of its mechanism (136). Furthermore, thalidomide derivatives, lenalidomide and pomalidomide, were developed for multiple myeloma (137, 138). These small molecules are known in the field as immunomodulatory drugs or “IMiDs.”

In 2010, through immunoprecipitation studies, the Handa group identified thalidomide as a ligand of the E3 ligase CRBN and recapitulated thalidomide's teratogenicity in zebrafish (139). Follow-up studies identified that thalidomide and its derivatives did not only inhibit the E3 ligase's interaction with its natural substrates but also caused a gain of function to induce degradation of zinc finger proteins including Ikaros (IKZF1) and Aiolos (IKZF3) (140, 141, 142). While not fully understood to date, much of the clinical effects of IMiDs are attributed to their ability to degrade neosubstrates. For instance, studies have now clarified that the teratogenicity initially observed was likely due to recruitment of the neosubstrate SALL4 (143).

Structural studies highlight that the glutarimide ring of the IMiD engages CRBN at its substrate-binding interface, in a tri-tryptophan pocket. The second half of the small molecule, the phthaloyl moiety, remains solvent exposed and acts as the warhead that recruits neosubstrates (140, 144). The ternary complex is further stabilized by protein–protein interactions between CRBN and the neosubstrate. The phthalimide ring of IMiDs is now understood to primarily promote the engagement of proteins that contain β-hairpin loops, and thus proteins that contain this secondary structure are in theory susceptible to degradation (144, 145). Interestingly, beyond a critical glycine, binding does not appear to be sequence-dependent. In fact, *in silico* proteomics studies have revealed up to 150 possible neosubstrates of IMiDs (145). In addition, small structural changes to IMiDs, which cause slight changes in neosubstrate affinity, can result in major differences in target selectivity within cells. For instance, while CK1α is a neosubstrate of lenalidomide, it is not degraded by other commonly used IMiDs (142). Like IMiDs, the non-IMiD anticancer agent indisulam causes G1/S cell cycle arrest by acting as a molecular glue for the E3 ligase DCAF15 and allows degradation of the neosubstrate RBM38 (146, 147).

More recent studies are now further expanding the possibilities of induced degradation by molecular glues. In a concerted effort, Slabicki *et al.* correlated drug sensitivity to mRNA levels of E3 ligase components to identify molecules that may act through a degradation mechanism of action (148). Through this effort they identified CR-8, a cyclin-dependent kinase (CDK) inhibitor, that induces selective degradation of cyclin K. Using genetic and stability reporter screens, they identified Cullin 4B, DDB1, RBX1, and neddylation machinery as essential for facilitating CR-8-induced degradation of cyclin K. However, they did not identify an established E3 substrate receptor such as DCAF proteins. Rather they showed that CDK12, which normally does not act as a E3 substrate receptor, binds DDB1 in the presence of CR-8, positioning cyclin K into the ubiquitination zone (radius from E3 ligase where accessible lysine residues on substrate can be marked with ubiquitin). Interestingly, similar results have now been published by the Winter (149) and Wang (150) groups. These studies show that other parts of the E3 ligase complex than the substrate recognition subunit may be amenable to hijacking for TPD. Furthermore, this study shows that it might be possible to reverse the IMiD mechanism of action and rather target a ligandable pocket on a specific POI and engage E3 ligases through small modifications.

Thus far, molecular glues are the only targeted protein degrader molecules that have been FDA-approved (thalidomide and its analogs). Generally, these molecules are smaller and obey the traditional “Lipinski rule of five,” which describes ideal properties for orally available drugs (151). In addition, molecular glues do not require a large hydrophobic pocket to engage a neosubstrate. However, studies have relied on phenotypic screens to identify molecular glues; there has been little progress in rational design of molecular glues. Therefore, despite their early establishment, much is to be learned for molecular glues to be as systematically generalizable as PROTACs.

## Co-opting the endolysosomal pathway with Lysosomal-Targeting Chimeras (LYTACs)

**Figure 3:**
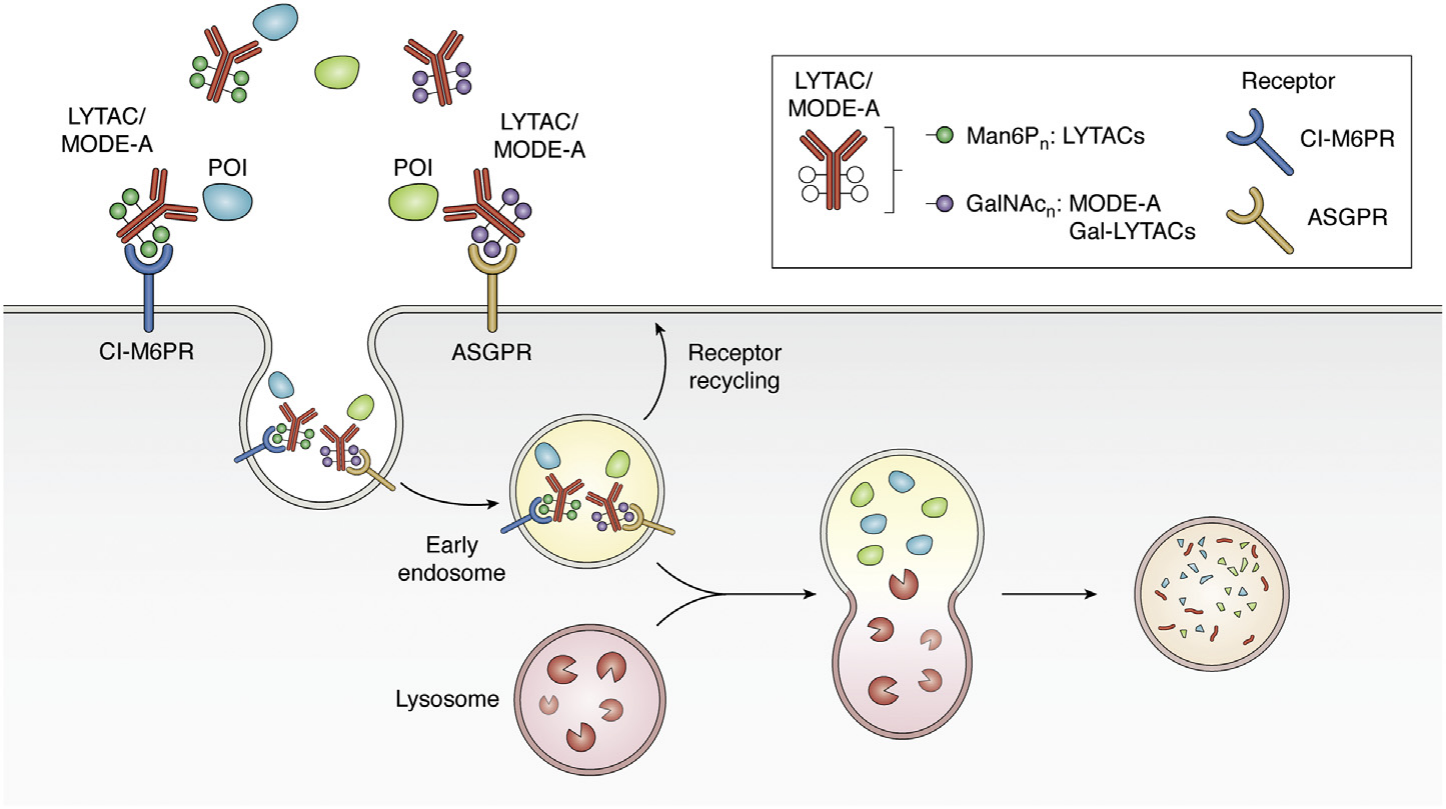
**Co-opting the endosomal–lysosome pathway with LYTACs and MoDE-As**. LYTACs and MoDE-As tether extracellular or membrane-bound POIs to a recycling receptor, which facilitates internalization and subsequent lysosomal degradation. LYTACs utilize the cation-independent mannose-6-phosphate receptor CI-M6PR, a recycling membrane protein that binds POIs labeled with an MP6n tag. GalNAc-LYTACs and MoDE-As utilize a GalNAC tag to recruit the asialoglycoprotein receptor (ASGPR), a liver-specific lysosomal targeting receptor, for tissue-specific degradation.

Extracellular proteins make up about 40% of the proteome and include important classes of proteins such as growth factors, cytokines, and more that have vital roles in disease progression (152, 153). PROTACs and molecular glues have been proven well adept at targeting proteins within the cell and in the cell membrane. However, due to the intracellular location of the UPS, proteins outside of the cell are inaccessible to PROTACs and molecular glues. To target extracellular proteins, the Bertozzi lab developed Lysosomal-Targeting Chimeras (LYTACs) (154) (Fig. 3).

In the first proof-of-concept study, the Bertozzi group utilized the cation-independent mannose-6-phosphate receptor (CI-M6PR), an important receptor in the trans-Golgi network, to co-opt the endocytic pathway (155). CI-M6PR binds mannose 6-phosphate (M6P) bearing proteins and Insulin-like growth factor 1 (IGF-I) from the extracellular space and targets them to the endosome and lysosome after internalization (156). The acidic pH of the lysosome triggers the release of the glycosylated cargo to be degraded by the lysosomal enzymes and acid hydrolases. The receptor is then shuttled back to the membrane to repeat the cycle (155, 156).

The Bertozzi group showed that by conjugating a synthetic oligopeptide ligand, mannose-6-phosphonate (M6Pn) to serine or lysine residues on antibodies, they can successfully induce their internalization and degradation (154). As a test case, they showed degradation of fluorescent streptavidin molecules using biotinylated MP6. Using this system, they also performed a CRISPR screen and identify that loss of the genes that encode proteins in the exocyst pathway as well as CI-M6PR abrogates LYTAC-induced degradation, establishing its mechanism of action. They confirm that the technology is amenable to target disease-causing extracellular proteins by targeting apolipoprotein E4 (ApoE4), a protein involved in Alzheimer's disease. Using M6Pn-functionalized cetuximab (monoclonal antibody against EGFR), they observe greater that 70% degradation of EGFR with short or long M6Pn chains. This occurs after 3 h but requires between 12 and 24 h to obtain maximal degradation. However, once degradation is achieved, it persists for at least 72 h. In addition, they show that PD-L1 can also be targeted *via* LYTACs, to cause 30–70% degradation depending on the antibody used. Thus, the LYTAC technique is amenable to induced degradation of extracellular and membrane-bound proteins.

In a similar vein, the Spiegel group recently released a preprint showcasing internalization and degradation of extracellular proteins in a system they termed MoDE-As (Molecular Degraders of Extracellular proteins through the Asialoglycoprotein receptor [ASGPR]) (157). ASGPR is a well-studied, highly expressed liver-specific receptor involved in internalization of several extracellular proteins. ASGPR binds galactose or N-acetyl galactosamine (GalNAc) and has been shown to turn over every 15 min (158). Their bifunctional small molecule, D-MoDE-As, is composed of an optimized trivalent GalNAc-based ligand, a PEG based linker, and a dinitrophenol (DNP) molecule. The authors showed that D-MoDe-As can successfully induce degradation of a DNP-binding antibody. Degradation occurs through induced ternary complex formation, followed by internalization and localization with LAMP2-positive late endosomes/lysosomes. They also show that MoDE-As can induce degradation of the extracellular protein MIF and cause its degradation *in vivo*.

The Tang and Bertozzi groups have similarly utilized ASGPR as the internalizing receptor for lysosomal induced degradation (159, 160). The Bertozzi group observed a higher internalization efficiency with ASGPR as compared with CI-M6PR, with decreases in surface EGFR occurring at tenfold lower concentrations. However, both cetuximab-GalNAC-LYTAC and cetuximab-M6Pn-LYTAC achieved similar levels of maximal degradation. Using specific integrin-binding peptide GalNAC (PIP-GalNAC), they show an increase in integrin depletion and a resultant enhancement of inhibition of cell proliferation. Lastly, they observed that a single site-specific tri-GalNAc ligand conjugated to their PIP protein caused its degradation. The Tang group also showed both small molecules (biotin) and antibodies (anti-biotin IgG-647) coupled to a tri-GalNAC can successfully induce internalization and degradation by the lysosome (159). In addition, their study showed that small proteins are more readily internalized than larger proteins.

Overall, these studies establish targeting of membrane and extracellular proteins to the lysosome for degradation as a possible therapeutic approach. Like the establishment of E3 ligase ligands in the PROTAC field, identification of additional recycling receptors will be important in propelling the field of LYTACs forward.

## Macroautophagy Degradation Targeting Chimeras (MADTACS): AUTACs/ATTECs

**Figure 4:**
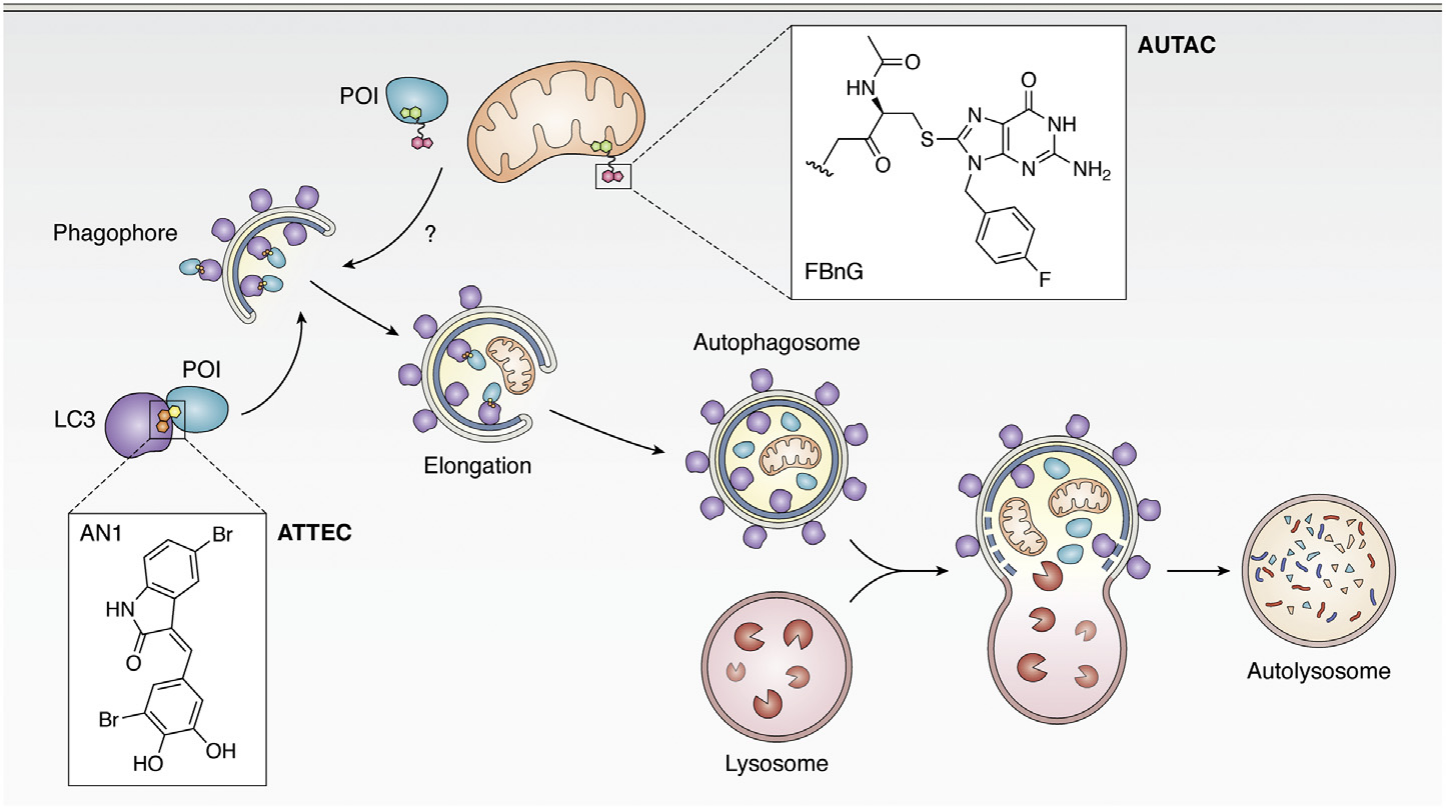
**Macroautophagy degradation targeting chimeras (MADTACs): AUTACs/ATTECs.** AUTACs encoporate FBnG, an autophagy-inducing molecule that mimics S-guanylation. Through an unknown mechanism, AUTACs trigger K63 ubiquitination and lysosomal degradation of POIs. ATTECs are small molecules that link mHTT to LC3, tethering the protein to a phagophore, and similarly induce degradation *via* autophagy.

Macroautophagy is an alternate degradation pathway within cells where lysosomes engulf and degrade cytosolic substrates (161). While the UPS primarily degrades proteins, macroautophagy can remove proteins, as well as larger objects such as dysfunctional organelles and intracellular pathogens (162). Macroautophagy is initiated by the nucleation of a phagophore, an isolation double membrane, by a complex of proteins including LC3-I (microtubules-associated protein light chain 3), the ULK complex, PI3K kinases, Beclin1, and p150 kinase (163). The phagophore then elongates and sequesters cytoplasm that contains the cargo to be degraded (164). During this process controlled by the Atg conjugation system, LC3-I is also conjugated to lipids on the elongating phagophore to form LC3-II (165). The membrane is then enclosed and matures to form an autophagosome, which then fuses with a lysosome to form an autolysosome (166). There, the acidic environment of the autolysosome and the hydrolases therein degrade the cargo (Fig. 4) (167).

S-guanylation (cysteine conjugation of 8-Nitro-cGMP) is a posttranslational modification important in xenophagy, an innate autoimmune response used to clear intracellular bacterial pathogens (168, 169). Upon escape from phagosomes, surface proteins on bacteria such as group A *streptococcus* are modified with 8-nitro-cGMP, causing K63 ubiquitination and lysosomal degradation of the microorganism. This process is Atg5-dependent and leads to accumulation of LC3-positive puncta in the cell (170). Beyond this observation, the exact mechanism by which S-guanylation triggers xenophagy is unknown (171).

In an elegant study, the Arimoto laboratory (that was instrumental in understanding the role of S-guanylation) showed that a cGMP tag alone is sufficient to induce selective autophagy (172). Similarly, to bacteria in xenophagy, HaloTag-GFP covalently labeled with cGMP localized with autophagosome markers such as LC3-II and K63 ubiquitin chains. Knockdown of important autophagy machinery such as Agt5 and p61/SqSTM-1 prevented localization with LC3-II and K63 ubiquitin, confirming the mechanism of action.

To avoid interfering with other natural pathways that use cGMP, the Arimoto group developed p-fluorobenzylguanine (FBnG) and showed that it similarly induced selective autophagy. With this autophagy initiator, they then created autophagy-targeting chimera (AUTAC-1), which similarly to the first PROTAC incorporated fumagillin to target MetAP2. AUTAC-1 induced ∼80% degradation of MetAP2 at concentrations greater than 1 μM. They also show that AUTACs are amenable to noncovalently induced degradation using the FKPB-targeting AUTAC, AUTAC-2. Due to the cytoplasmic restriction of lysosomes, nuclear protein BRD4 degradation with a JQ-1-based AUTAC was low (∼30%) and likely only occurred during cell division.

The Arimoto group further showed that FBnG tagging is also capable of inducing degradation of mitochondria by expressing a fusion protein of outer mitochondrial membrane (OMM) protein 25 and EGFP-HT and targeting it for degradation using a FBnG-Halotag ligand (∼50% degradation at concentrations >10 μM after 12 h). Using this system, they demonstrate that AUTACs can be used for nonprotein substrates. Lastly, using a mitochondrial translocator protein (TSPO), they show that they can induce mitophagy in a dysfunctional mitochondrial system.

In a more directed approach, the Lu group performed a microarray screen for compounds that dually engage LC3 and mutant huntingtin protein (mHTT) but not WT huntingtin protein. Through this screen and counter screen, they identified two compounds (10O5 and 8F20) that simultaneously bind LC3 and mHTT (173, 174). In primary HD knock-in mouse models, both compounds induced mutant-selective degradation of mHTT.

Both compounds are relatively small (<500 kDa) and contain a common pharmacophore of an aryl ring connected to a lactam-based bicyclic structure. Indeed, by testing compounds with similar features, they identified AN1 and AN2, which also induced mHTT-selective degradation. Their mechanistic studies reveal that mutant selectivity is achieved through binding of the polyglutamine-repeat region on mHTT that is missing from WT HTT. 10O5, AN1, AN2 induced a hook effect, implying that these molecules can independently interact with LC3 and mHTT despite their small size.

Like AUTACs, ATTECs require Atg5 activity and localize to LC3-II and autophagosomes. Furthermore, inhibition of lysosomal halts the ATTEC-induced degradation confirming that loss of mHTT occurs *via* autophagy. Lastly, they show that degradation of mHTT can be observed with 0.5 mg/kg *in vivo* in mice and fly models with no toxicity or targeting of WT HTT. Using AUTACs and ATTECs, the Hashimoto and Lu groups both have shown that autophagy can be co-opted to induce degradation of POIs.

The AUTAC strategy in theory allows targeting of all within the cell; however, the exact mechanism of action is unknown. In fact, it is quite opportune that the Arimoto group was able to make structural changes to develop FnBG without abrogating the autophagy-inducing ability of the compound. Despite this, AUTAC-mediated degradation is not potent, at times requiring high micromolar concentrations. Thus, identifying the target of FnBG will allow for better understanding of the technology as well as allow for more potent AUTACs to be created.

Conversely, ATTECs induce highly selective degradation of their target, and the molecular target of the small molecules has been elucidated. However, ATTECs are limited in the breadth of targets and are selective only for proteins with polyarginine groups. Much biophysical/structural studies must be performed to establish the LC3-binding moiety to create ATTECs that induce degradation of other proteins.

## Summary and perspective

### Beyond protein degradation

The TPD field has expanded the possibility of how protein function can be modulated by small molecules. It has also encouraged others to “think outside of the box” on innovative ways that macromolecule function can be modulated. Within the past few years, strategies have been developed to induce the deubiquitination of POIs (175). Beyond this, scientists have used chimeric molecules to induce phosphorylation (Phosphorylation-Inducing Chimeras, PHIC) (176). In a reverse fashion, chimeric molecules have been engineered to dephosphorylate kinases implicated in cancer (177, 178). Beyond proteins, bifunctional molecules have also been used to target microRNAs implicated in disease for degradation with ribonucleases (11).

Over the past 20 years, chemical biologists have used small molecules to control the fate of disease-causing proteins. While PROTACs and molecular glues primarily shuttle proteins to the proteosome, LYTACs and AUTACs/ATTECs utilize the lysosome. Each technology is primed for removing proteins from specific spaces and together comprising a toolbox that one can draw from to appropriately match a target protein.

Though slow to gain traction, today TPD is accepted as a major modality in the academic and pharmaceutical space. Chemical biologists capitalized on the understanding of the degradation pathways to remove proteins from inside and outside of cells. In a cyclical fashion, the TPD field is now contributing to the knowledge base by revealing novel protein functions.

Orally bioavailable PROTACs have now entered clinical trials and are showing promise in castration-resistant prostate cancer (ARV-110) and ER+/HER2-breast cancer (ARV-471). All eyes lie on these clinical candidates as they pave the way for future drugs of this class. Overall, this field is in its formative years with much to be learned and even more to be discovered.

## Conflict of interest

C. M. C. is founder, shareholder, and consultant to Arvinas, Inc and Halda, LLC, which support research in his laboratory.

## References

[1] Balch W.E., Morimoto R.I., Dillin A., Kelly J.W. (2008). Adapting proteostasis for disease intervention. Science.

[2] Adhikari B., Bozilovic J., Diebold M., Schwarz J.D., Hofstetter J., Schröder M., Wanior M., Narain A., Vogt M., Dudvarski Stankovic N., Baluapuri A., Schönemann L., Eing L., Bhandare P., Kuster B. (2020). PROTAC-mediated degradation reveals a non-catalytic function of AURORA-A kinase. Nat. Chem. Biol..

[3] Powers E.T., Morimoto R.I., Dillin A., Kelly J.W., Balch W.E. (2009). Biological and chemical approaches to diseases of proteostasis deficiency. Annu. Rev. Biochem..

[4] Chen B., Retzlaff M., Roos T., Frydman J. (2011). Cellular strategies of protein quality control. Cold Spring Harb. Perspect. Biol..

[5] Dissmeyer N., Coux O., Rodriguez M.S., Barrio R. (2019). Proteostasis: A European network to break barriers and integrate science on protein homeostasis. Trends Biochem. Sci..

[6] Hershko A., Ciechanover A. (1998). The ubiquitin system. Annu. Rev. Biochem..

[7] Dantuma N.P., Bott L.C. (2014). The ubiquitin-proteasome system in neurodegenerative diseases: Precipitating factor, yet part of the solution. Front. Mol. Neurosci..

[8] Kleiger G., Mayor T. (2014). Perilous journey: A tour of the ubiquitin–proteasome system. Trends Cell Biol..

[9] Komander D., Rape M. (2012). The ubiquitin code. Annu. Rev. Biochem..

[10] Metzger M.B., Hristova V.A., Weissman A.M. (2012). HECT and RING finger families of E3 ubiquitin ligases at a glance. J. Cell Sci..

[11] Costales M.G., Aikawa H., Li Y., Childs-Disney J.L., Abegg D., Hoch D.G., Pradeep Velagapudi S., Nakai Y., Khan T., Wang K.W., Yildirim I., Adibekian A., Wang E.T., Disney M.D. (2020). Small-molecule targeted recruitment of a nuclease to cleave an oncogenic RNA in a mouse model of metastatic cancer. Proc. Natl. Acad. Sci. U. S. A..

[12] Sakamoto K.M., Kim K.B., Kumagai A., Mercurio F., Crews C.M., Deshaies R.J. (2001). Protacs: Chimeric molecules that target proteins to the Skp1-Cullin-F box complex for ubiquitination and degradation. Proc. Natl. Acad. Sci. U. S. A..

[13] Sakamoto K.M., Kim K.B., Verma R., Ransick A., Stein B., Crews C.M., Deshaies R.J. (2003). Development of Protacs to target cancer-promoting proteins for ubiquitination and degradation. Mol. Cell Proteomics.

[14] Schneekloth J.S., Fonseca F.N., Koldobskiy M., Mandal A., Deshaies R., Sakamoto K., Crews C.M. (2004). Chemical genetic control of protein levels: Selective *in vivo* targeted degradation. J. Am. Chem. Soc..

[15] Schneekloth A.R., Pucheault M., Tae H.S., Crews C.M. (2008). Targeted intracellular protein degradation induced by a small molecule: En route to chemical proteomics. Bioorg. Med. Chem. Lett..

[16] Bai L., Zhou H., Xu R., Zhao Y., Chinnaswamy K., McEachern D., Chen J., Yang C.Y., Liu Z., Wang M., Liu L., Jiang H., Wen B., Kumar P., Meagher J.L. (2019). A potent and selective small-molecule degrader of STAT3 achieves complete tumor regression in vivo. Cancer Cell.

[17] Burslem G.M., Smith B.E., Lai A.C., Jaime-Figueroa S., McQuaid D.C., Bondeson D.P., Toure M., Dong H., Qian Y., Wang J., Crew A.P., Hines J., Crews C.M. (2018). The advantages of targeted protein degradation over inhibition: An RTK case study. Cell Chem. Biol..

[18] Buhimschi A.D., Armstrong H.A., Toure M., Jaime-Figueroa S., Chen T.L., Lehman A.M., Woyach J.A., Johnson A.J., Byrd J.C., Crews C.M. (2018). Targeting the C481S ibrutinib-resistance mutation in Bruton's tyrosine kinase using PROTAC-mediated degradation. Biochemistry.

[19] Pettersson M., Crews C.M. (2019). PROteolysis TArgeting Chimeras (PROTACs) - past, present and future. Drug Discov. Today Technol..

[20] Lu M., Liu T., Jiao Q., Ji J., Tao M., Liu Y., You Q., Jiang Z. (2018). Discovery of a Keap1-dependent peptide PROTAC to knockdown Tau by ubiquitination-proteasome degradation pathway. Eur. J. Med. Chem..

[21] Kargbo R.B. (2019). Treatment of Alzheimer's by PROTAC-tau protein degradation. ACS Med. Chem. Lett..

[22] Kargbo R.B. (2020). PROTAC-mediated degradation of KRAS protein for anticancer therapeutics. ACS Med. Chem. Lett..

[23] Zeng M., Xiong Y., Safaee N., Nowak R.P., Donovan K.A., Yuan C.J., Nabet B., Gero T.W., Feru F., Li L., Gondi S., Ombelets L.J., Quan C., Jänne P.A., Kostic M. (2020). Exploring targeted degradation strategy for oncogenic KRAS(G12C). Cell Chem. Biol..

[24] Bond M.J., Chu L., Nalawansha D.A., Li K., Crews C.M. (2020). Targeted degradation of oncogenic KRASG12C by VHL-recruiting PROTACs. ACS Cent. Sci..

[25] Buckley D.L., Raina K., Darricarrere N., Hines J., Gustafson J.L., Smith I.E., Miah A.H., Harling J.D., Crews C.M. (2015). HaloPROTACS: Use of small molecule PROTACs to induce degradation of HaloTag fusion proteins. ACS Chem. Biol..

[26] Nabet B., Ferguson F.M., Seong B.K.A., Kuljanin M., Leggett A.L., Mohardt M.L., Robichaud A., Conway A.S., Buckley D.L., Mancias J.D., Bradner J.E., Stegmaier K., Gray N.S. (2020). Rapid and direct control of target protein levels with VHL-recruiting dTAG molecules. Nat. Commun..

[27] Nabet B., Roberts J.M., Buckley D.L., Paulk J., Dastjerdi S., Yang A., Leggett A.L., Erb M.A., Lawlor M.A., Souza A., Scott T.G., Vittori S., Perry J.A., Qi J., Winter G.E. (2018). The dTAG system for immediate and target-specific protein degradation. Nat. Chem. Biol..

[28] Sun X., Gao H., Yang Y., He M., Wu Y., Song Y., Tong Y., Rao Y. (2019). PROTACs: Great opportunities for academia and industry. Signal Transduct. Target. Ther..

[29] Nalawansha D.A., Crews C.M. (2020). PROTACs: An emerging therapeutic modality in precision medicine. Cell Chem. Biol..

[30] Burslem G.M., Crews C.M. (2020). Proteolysis-targeting chimeras as therapeutics and tools for biological discovery. Cell.

[31] Iconomou M., Saunders D.N. (2016). Systematic approaches to identify E3 ligase substrates. Biochem. J..

[32] LeBlanc N., Mallette E., Zhang W. (2021). Targeted modulation of E3 ligases using engineered ubiquitin variants. FEBS J..

[33] Orlicky S., Tang X., Neduva V., Elowe N., Brown E.D., Sicheri F., Tyers M. (2010). An allosteric inhibitor of substrate recognition by the SCFCdc4 ubiquitin ligase. Nat. Biotechnol..

[34] Aghajan M., Jonai N., Flick K., Fu F., Luo M., Cai X., Ouni I., Pierce N., Tang X., Lomenick B., Damoiseaux R., Hao R., Del Moral P.M., Verma R., Li Y. (2010). Chemical genetics screen for enhancers of rapamycin identifies a specific inhibitor of an SCF family E3 ubiquitin ligase. Nat. Biotechnol..

[35] Lydeard J.R., Harper J.W. (2010). Inhibitors for E3 ubiquitin ligases. Nat. Biotechnol..

[36] Vassilev L.T., Vu B.T., Graves B., Carvajal D., Podlaski F., Filipovic Z., Kong N., Kammlott U., Lukacs C., Klein C., Fotouhi N., Liu E.A. (2004). *In Vivo* activation of the p53 pathway by small-molecule antagonists of MDM2. Science.

[37] Itoh Y., Ishikawa M., Naito M., Hashimoto Y. (2010). Protein knockdown using methyl Bestatin−Ligand hybrid molecules: Design and synthesis of inducers of ubiquitination-mediated degradation of cellular retinoic acid-binding proteins. J. Am. Chem. Soc..

[38] Deveraux Q.L., Reed J.C. (1999). IAP family proteins--suppressors of apoptosis. Genes Dev..

[39] Okuhira K., Ohoka N., Sai K., Nishimaki-Mogami T., Itoh Y., Ishikawa M., Hashimoto Y., Naito M. (2011). Specific degradation of CRABP-II via cIAP1-mediated ubiquitylation induced by hybrid molecules that crosslink cIAP1 and the target protein. FEBS Lett..

[40] Okuhira K., Demizu Y., Hattori T., Ohoka N., Shibata N., Kurihara M., Naito M. (2016). Molecular design, synthesis, and evaluation of SNIPER(ER) that induces proteasomal degradation of ERα. Methods Mol. Biol..

[41] Ohoka N., Shibata N., Hattori T., Naito M. (2016). Protein knockdown technology: Application of ubiquitin ligase to cancer therapy. Curr. Cancer Drug Targets.

[42] Ohoka N., Nagai K., Hattori T., Okuhira K., Shibata N., Cho N., Naito M. (2014). Cancer cell death induced by novel small molecules degrading the TACC3 protein via the ubiquitin-proteasome pathway. Cell Death Dis..

[43] Itoh Y., Kitaguchi R., Ishikawa M., Naito M., Hashimoto Y. (2011). Design, synthesis and biological evaluation of nuclear receptor-degradation inducers. Bioorg. Med. Chem..

[44] Itoh Y., Ishikawa M., Kitaguchi R., Sato S., Naito M., Hashimoto Y. (2011). Development of target protein-selective degradation inducer for protein knockdown. Bioorg. Med. Chem..

[45] Fan X., Wang Y.T. (2015). SNIPER peptide-mediated degradation of endogenous proteins. Curr. Protoc. Chem. Biol..

[46] Demizu Y., Shibata N., Hattori T., Ohoka N., Motoi H., Misawa T., Shoda T., Naito M., Kurihara M. (2016). Development of BCR-ABL degradation inducers via the conjugation of an imatinib derivative and a cIAP1 ligand. Bioorg. Med. Chem. Lett..

[47] Okuhira K., Shoda T., Omura R., Ohoka N., Hattori T., Shibata N., Demizu Y., Sugihara R., Ichino A., Kawahara H., Itoh Y., Ishikawa M., Hashimoto Y., Kurihara M., Itoh S. (2017). Targeted degradation of proteins localized in subcellular compartments by hybrid small molecules. Mol. Pharmacol..

[48] Shibata N., Miyamoto N., Nagai K., Shimokawa K., Sameshima T., Ohoka N., Hattori T., Imaeda Y., Nara H., Cho N., Naito M. (2017). Development of protein degradation inducers of oncogenic BCR-ABL protein by conjugation of ABL kinase inhibitors and IAP ligands. Cancer Sci..

[49] Ohoka N., Ujikawa O., Shimokawa K., Sameshima T., Shibata N., Hattori T., Nara H., Cho N., Naito M. (2019). Different degradation mechanisms of inhibitor of apoptosis proteins (IAPs) by the specific and nongenetic IAP-dependent protein eraser (SNIPER). Chem. Pharm. Bull (Tokyo).

[50] Ohoka N., Okuhira K., Ito M., Nagai K., Shibata N., Hattori T., Ujikawa O., Shimokawa K., Sano O., Koyama R., Fujita H., Teratani M., Matsumoto H., Imaeda Y., Nara H. (2017). *In vivo* knockdown of pathogenic proteins via specific and nongenetic inhibitor of apoptosis protein (IAP)-dependent protein Erasers (SNIPERs). J. Biol. Chem..

[51] Ohoka N., Morita Y., Nagai K., Shimokawa K., Ujikawa O., Fujimori I., Ito M., Hayase Y., Okuhira K., Shibata N., Hattori T., Sameshima T., Sano O., Koyama R., Imaeda Y. (2018). Derivatization of inhibitor of apoptosis protein (IAP) ligands yields improved inducers of estrogen receptor α degradation. J. Biol. Chem..

[52] Deveraux Q.L., Takahashi R., Salvesen G.S., Reed J.C. (1997). X-linked IAP is a direct inhibitor of cell-death proteases. Nature.

[53] Buckley D.L., Gustafson J.L., Van Molle I., Roth A.G., Tae H.S., Gareiss P.C., Jorgensen W.L., Ciulli A., Crews C.M. (2012). Small-molecule inhibitors of the interaction between the E3 ligase VHL and HIF1alpha. Angew. Chem. Int. Ed. Engl..

[54] Buckley D.L., Van Molle I., Gareiss P.C., Tae H.S., Michel J., Noblin D.J., Jorgensen W.L., Ciulli A., Crews C.M. (2012). Targeting the von Hippel-Lindau E3 ubiquitin ligase using small molecules to disrupt the VHL/HIF-1alpha interaction. J. Am. Chem. Soc..

[55] Rodriguez-Gonzalez A., Cyrus K., Salcius M., Kim K., Crews C.M., Deshaies R.J., Sakamoto K.M. (2008). Targeting steroid hormone receptors for ubiquitination and degradation in breast and prostate cancer. Oncogene.

[56] Buckley D.L., Crews C.M. (2014). Small-molecule control of intracellular protein levels through modulation of the ubiquitin proteasome system. Angew. Chem. Int. Ed. Engl..

[57] Maniaci C., Hughes S.J., Testa A., Chen W., Lamont D.J., Rocha S., Alessi D.R., Romeo R., Ciulli A. (2017). Homo-PROTACs: Bivalent small-molecule dimerizers of the VHL E3 ubiquitin ligase to induce self-degradation. Nat. Commun..

[58] Gadd M.S., Testa A., Lucas X., Chan K.H., Chen W., Lamont D.J., Zengerle M., Ciulli A. (2017). Structural basis of PROTAC cooperative recognition for selective protein degradation. Nat. Chem. Biol..

[59] Bondeson D.P., Smith B.E., Burslem G.M., Buhimschi A.D., Hines J., Jaime-Figueroa S., Wang J., Hamman B.D., Ishchenko A., Crews C.M. (2018). Lessons in PROTAC design from selective degradation with a promiscuous warhead. Cell Chem. Biol..

[60] Testa A., Lucas X., Castro G.V., Chan K.H., Wright J.E., Runcie A.C., Gadd M.S., Harrison W.T.A., Ko E.J., Fletcher D., Ciulli A. (2018). 3-Fluoro-4-hydroxyprolines: Synthesis, conformational analysis, and stereoselective recognition by the VHL E3 ubiquitin ligase for targeted protein degradation. J. Am. Chem. Soc..

[61] Zhang L., Riley-Gillis B., Vijay P., Shen Y. (2019). Acquired resistance to BET-PROTACs (Proteolysis-Targeting chimeras) caused by genomic alterations in core components of E3 ligase complexes. Mol. Cancer Ther..

[62] Khan S., Zhang X., Lv D., Zhang Q., He Y., Zhang P., Liu X., Thummuri D., Yuan Y., Wiegand J.S., Pei J., Zhang W., Sharma A., McCurdy C.R., Kuruvilla V.M. (2019). A selective BCL-XL PROTAC degrader achieves safe and potent antitumor activity. Nat. Med..

[63] He Y., Koch R., Budamagunta V., Zhang P., Zhang X., Khan S., Thummuri D., Ortiz Y.T., Zhang X., Lv D., Wiegand J.S., Li W., Palmer A.C., Zheng G., Weinstock D.M. (2020). DT2216-a Bcl-xL-specific degrader is highly active against Bcl-xL-dependent T cell lymphomas. J. Hematol. Oncol..

[64] Ito T., Ando H., Suzuki T., Ogura T., Hotta K., Imamura Y., Yamaguchi Y., Handa H. (2010). Identification of a primary target of thalidomide teratogenicity. Science.

[65] Robb C.M., Contreras J.I., Kour S., Taylor M.A., Abid M., Sonawane Y.A., Zahid M., Murry D.J., Natarajan A., Rana S. (2017). Chemically induced degradation of CDK9 by a proteolysis targeting chimera (PROTAC). Chem. Commun. (Camb).

[66] Burslem G.M., Ottis P., Jaime-Figueroa S., Morgan A., Cromm P.M., Toure M., Crews C.M. (2018). Efficient synthesis of immunomodulatory drug analogues enables exploration of structure-degradation relationships. ChemMedChem.

[67] Zorba A., Nguyen C., Xu Y., Starr J., Borzilleri K., Smith J., Zhu H., Farley K.A., Ding W., Schiemer J., Feng X., Chang J.S., Uccello D.P., Young J.A., Garcia-Irrizary C.N. (2018). Delineating the role of cooperativity in the design of potent PROTACs for BTK. Proc. Natl. Acad. Sci. U. S. A..

[68] Minko T. (2020). Nanoformulation of BRD4-degrading PROTAC: Improving druggability to target the ‘undruggable’ MYC in pancreatic cancer. Trends Pharmacol. Sci..

[69] Lai A.C., Toure M., Hellerschmied D., Salami J., Jaime-Figueroa S., Ko E., Hines J., Crews C.M. (2016). Modular PROTAC design for the degradation of oncogenic BCR-ABL. Angew. Chem. Int. Ed. Engl..

[70] Ishoey M., Chorn S., Singh N., Jaeger M.G., Brand M., Paulk J., Bauer S., Erb M.A., Parapatics K., Müller A.C., Bennett K.L., Ecker G.F., Bradner J.E., Winter G.E. (2018). Translation termination factor GSPT1 is a phenotypically relevant off-target of heterobifunctional phthalimide degraders. ACS Chem. Biol..

[71] Ottis P., Toure M., Cromm P.M., Ko E., Gustafson J.L., Crews C.M. (2017). Assessing different E3 ligases for small molecule induced protein ubiquitination and degradation. ACS Chem. Biol..

[72] Ward C.C., Kleinman J.I., Brittain S.M., Lee P.S., Chung C.Y.S., Kim K., Petri Y., Thomas J.R., Tallarico J.A., McKenna J.M., Schirle M., Nomura D.K. (2019). Covalent ligand screening uncovers a RNF4 E3 ligase recruiter for targeted protein degradation applications. ACS Chem. Biol..

[73] Zhang X., Crowley V.M., Wucherpfennig T.G., Dix M.M., Cravatt B.F. (2019). Electrophilic PROTACs that degrade nuclear proteins by engaging DCAF16. Nat. Chem. Biol..

[74] Tong B., Luo M., Xie Y., Spradlin J.N., Tallarico J.A., McKenna J.M., Schirle M., Maimone T.J., Nomura D.K. (2020). Bardoxolone conjugation enables targeted protein degradation of BRD4. Sci. Rep..

[75] Tong B., Spradlin J.N., Novaes L.F.T., Zhang E., Hu X., Moeller M., Brittain S.M., McGregor L.M., McKenna J.M., Tallarico J.A., Schirle M., Maimone T.J., Nomura D.K. (2020). A nimbolide-based kinase degrader preferentially degrades oncogenic BCR-ABL. ACS Chem. Biol..

[76] Spradlin J.N., Hu X., Ward C.C., Brittain S.M., Jones M.D., Ou L., To M., Proudfoot A., Ornelas E., Woldegiorgis M., Olzmann J.A., Bussiere D.E., Thomas J.R., Tallarico J.A., McKenna J.M. (2019). Harnessing the anti-cancer natural product nimbolide for targeted protein degradation. Nat. Chem. Biol..

[77] Sakamoto K.M. (2005). Chimeric molecules to target proteins for ubiquitination and degradation. Methods Enzymol.

[78] He X., Da Ros S., Nelson J., Zhu X., Jiang T., Okram B., Jiang S., Michellys P.-Y., Iskandar M., Espinola S., Jia Y., Bursulaya B., Kreusch A., Gao M.-Y., Spraggon G. (2017). Identification of potent and selective RIPK2 inhibitors for the treatment of inflammatory diseases. ACS Med. Chem. Lett..

[79] Bondeson D.P., Mares A., Smith I.E., Ko E., Campos S., Miah A.H., Mulholland K.E., Routly N., Buckley D.L., Gustafson J.L., Zinn N., Grandi P., Shimamura S., Bergamini G., Faelth-Savitski M. (2015). Catalytic *in vivo* protein knockdown by small-molecule PROTACs. Nat. Chem. Biol..

[80] Salami J., Alabi S., Willard R.R., Vitale N.J., Wang J., Dong H., Jin M., McDonnell D.P., Crew A.P., Neklesa T.K., Crews C.M. (2018). Androgen receptor degradation by the proteolysis-targeting chimera ARCC-4 outperforms enzalutamide in cellular models of prostate cancer drug resistance. Commun. Biol..

[81] Lu J., Qian Y., Altieri M., Dong H., Wang J., Raina K., Hines J., Winkler J.D., Crew A.P., Coleman K., Crews C.M. (2015). Hijacking the E3 ubiquitin ligase cereblon to efficiently target BRD4. Chem. Biol..

[82] Crew A.P., Raina K., Dong H., Qian Y., Wang J., Vigil D., Serebrenik Y.V., Hamman B.D., Morgan A., Ferraro C., Siu K., Neklesa T.K., Winkler J.D., Coleman K.G., Crews C.M. (2018). Identification and characterization of von Hippel-Lindau-recruiting proteolysis targeting chimeras (PROTACs) of TANK-binding kinase 1. J. Med. Chem..

[83] Roy M.J., Winkler S., Hughes S.J., Whitworth C., Galant M., Farnaby W., Rumpel K., Ciulli A. (2019). SPR-measured dissociation kinetics of PROTAC ternary complexes influence target degradation rate. ACS Chem. Biol..

[84] Noblejas-Lopez M.D.M., Nieto-Jimenez C., Burgos M., Gomez-Juarez M., Montero J.C., Esparis-Ogando A., Pandiella A., Galan-Moya E.M., Ocana A. (2019). Activity of BET-proteolysis targeting chimeric (PROTAC) compounds in triple negative breast cancer. J. Exp. Clin. Cancer Res..

[85] Maneiro M.A., Forte N., Shchepinova M.M., Kounde C.S., Chudasama V., Baker J.R., Tate E.W. (2020). Antibody-PROTAC conjugates enable HER2-dependent targeted protein degradation of BRD4. ACS Chem. Biol..

[86] Sun B., Fiskus W., Qian Y., Rajapakshe K., Raina K., Coleman K.G., Crew A.P., Shen A., Saenz D.T., Mill C.P., Nowak A.J., Jain N., Zhang L., Wang M., Khoury J.D. (2018). BET protein proteolysis targeting chimera (PROTAC) exerts potent lethal activity against mantle cell lymphoma cells. Leukemia.

[87] Lu Q., Ding X., Huang T., Zhang S., Li Y., Xu L., Chen G., Ying Y., Wang Y., Feng Z., Wang L., Zou X. (2019). BRD4 degrader ARV-825 produces long-lasting loss of BRD4 protein and exhibits potent efficacy against cholangiocarcinoma cells. Am. J. Transl. Res..

[88] Rathod D., Fu Y., Patel K. (2019). BRD4 PROTAC as a novel therapeutic approach for the treatment of vemurafenib resistant melanoma: Preformulation studies, formulation development and *in vitro* evaluation. Eur. J. Pharm. Sci..

[89] Pervaiz M., Mishra P., Günther S. (2018). Bromodomain drug discovery - the past, the present, and the future. Chem. Rec..

[90] Zhang F., Wu Z., Chen P., Zhang J., Wang T., Zhou J., Zhang H. (2020). Discovery of a new class of PROTAC BRD4 degraders based on a dihydroquinazolinone derivative and lenalidomide/pomalidomide. Bioorg. Med. Chem..

[91] Abruzzese M.P., Bilotta M.T., Fionda C., Zingoni A., Soriani A., Vulpis E., Borrelli C., Zitti B., Petrucci M.T., Ricciardi M.R., Molfetta R., Paolini R., Santoni A., Cippitelli M. (2016). Inhibition of bromodomain and extra-terminal (BET) proteins increases NKG2D ligand MICA expression and sensitivity to NK cell-mediated cytotoxicity in multiple myeloma cells: Role of cMYC-IRF4-miR-125b interplay. J. Hematol. Oncol..

[92] Xue G., Wang K., Zhou D., Zhong H., Pan Z. (2019). Light-induced protein degradation with photocaged PROTACs. J. Am. Chem. Soc..

[93] Hines J., Lartigue S., Dong H., Qian Y., Crews C.M. (2019). MDM2-Recruiting PROTAC offers superior, synergistic antiproliferative activity via simultaneous degradation of BRD4 and stabilization of p53. Cancer Res..

[94] Saenz D.T., Fiskus W., Qian Y., Manshouri T., Rajapakshe K., Raina K., Coleman K.G., Crew A.P., Shen A., Mill C.P., Sun B., Qiu P., Kadia T.M., Pemmaraju N., DiNardo C. (2017). Novel BET protein proteolysis-targeting chimera exerts superior lethal activity than bromodomain inhibitor (BETi) against post-myeloproliferative neoplasm secondary (s) AML cells. Leukemia.

[95] Raina K., Lu J., Qian Y., Altieri M., Gordon D., Rossi A.M., Wang J., Chen X., Dong H., Siu K., Winkler J.D., Crew A.P., Crews C.M., Coleman K.G. (2016). PROTAC-induced BET protein degradation as a therapy for castration-resistant prostate cancer. Proc. Natl. Acad. Sci. U. S. A..

[96] Fu Y., Rathod D., Patel K. (2020). Protein kinase C inhibitor anchored BRD4 PROTAC PEGylated nanoliposomes for the treatment of vemurafenib-resistant melanoma. Exp. Cell Res..

[97] DeMars K.M., Yang C., Castro-Rivera C.I., Candelario-Jalil E. (2018). Selective degradation of BET proteins with dBET1, a proteolysis-targeting chimera, potently reduces pro-inflammatory responses in lipopolysaccharide-activated microglia. Biochem. Biophys. Res. Commun..

[98] Yang C.Y., Qin C., Bai L., Wang S. (2019). Small-molecule PROTAC degraders of the bromodomain and extra terminal (BET) proteins - a review. Drug Discov. Today Technol..

[99] Testa A., Hughes S.J., Lucas X., Wright J.E., Ciulli A. (2020). Structure-based design of a macrocyclic PROTAC. Angew. Chem. Int. Ed. Engl..

[100] Duan Y., Guan Y., Qin W., Zhai X., Yu B., Liu H. (2018). Targeting Brd4 for cancer therapy: Inhibitors and degraders. Medchemcomm.

[101] Qin A.C., Jin H., Song Y., Gao Y., Chen Y.F., Zhou L.N., Wang S.S., Lu X.S. (2020). The therapeutic effect of the BRD4-degrading PROTAC A1874 in human colon cancer cells. Cell Death Dis..

[102] Riching K.M., Mahan S., Corona C.R., McDougall M., Vasta J.D., Robers M.B., Urh M., Daniels D.L. (2018). Quantitative live-cell kinetic degradation and mechanistic profiling of PROTAC mode of action. ACS Chem. Biol..

[103] Smith B.E., Wang S.L., Jaime-Figueroa S., Harbin A., Wang J., Hamman B.D., Crews C.M. (2019). Differential PROTAC substrate specificity dictated by orientation of recruited E3 ligase. Nat. Commun..

[104] Zoppi V., Hughes S.J., Maniaci C., Testa A., Gmaschitz T., Wieshofer C., Koegl M., Riching K.M., Daniels D.L., Spallarossa A., Ciulli A. (2019). Iterative design and optimization of initially inactive proteolysis targeting chimeras (PROTACs) identify VZ185 as a potent, fast, and selective von Hippel-Lindau (VHL) based dual degrader probe of BRD9 and BRD7. J. Med. Chem..

[105] Chung C.-w., Dai H., Fernandez E., Tinworth C.P., Churcher I., Cryan J., Denyer J., Harling J.D., Konopacka A., Queisser M.A., Tame C.J., Watt G., Jiang F., Qian D., Benowitz A.B. (2020). Structural insights into PROTAC-mediated degradation of Bcl-xL. ACS Chem. Biol..

[106] Kale J., Osterlund E.J., Andrews D.W. (2018). BCL-2 family proteins: Changing partners in the dance towards death. Cell Death Differ..

[107] Souers A.J., Leverson J.D., Boghaert E.R., Ackler S.L., Catron N.D., Chen J., Dayton B.D., Ding H., Enschede S.H., Fairbrother W.J., Huang D.C., Hymowitz S.G., Jin S., Khaw S.L., Kovar P.J. (2013). ABT-199, a potent and selective BCL-2 inhibitor, achieves antitumor activity while sparing platelets. Nat. Med..

[108] Drummond M.L., Williams C.I. (2019). In silico modeling of PROTAC-mediated ternary complexes: Validation and application. J. Chem. Inf. Model.

[109] Zaidman D., Prilusky J., London N. (2020). PRosettaC: Rosetta based modeling of PROTAC mediated ternary complexes. J. Chem. Inf. Model.

[110] Drummond M.L., Henry A., Li H., Williams C.I. (2020). Improved accuracy for modeling PROTAC-mediated ternary complex formation and targeted protein degradation via new in silico methodologies. J. Chem. Inf. Model..

[111] Bartlett D.W., Gilbert A.M. (2020). A kinetic proofreading model for bispecific protein degraders. J. Pharmacokinet. Pharmacodyn..

[112] Posternak G., Tang X., Maisonneuve P., Jin T., Lavoie H., Daou S., Orlicky S., Goullet de Rugy T., Caldwell L., Chan K., Aman A., Prakesch M., Poda G., Mader P., Wong C. (2020). Functional characterization of a PROTAC directed against BRAF mutant V600E. Nat. Chem. Biol..

[113] Alabi S., Jaime-Figueroa S., Yao Z., Gao Y., Hines J., Samarasinghe K.T.G., Vogt L., Rosen N., Crews C.M. (2021). Mutant-selective degradation by BRAF-targeting PROTACs. Nat. Commun.

[114] He Y., Zhang X., Chang J., Kim H.N., Zhang P., Wang Y., Khan S., Liu X., Zhang X., Lv D., Song L., Li W., Thummuri D., Yuan Y., Wiegand J.S. (2020). Using proteolysis-targeting chimera technology to reduce navitoclax platelet toxicity and improve its senolytic activity. Nat. Commun..

[115] Zhang X., He Y., Zhang P., Budamagunta V., Lv D., Thummuri D., Yang Y., Pei J., Yuan Y., Zhou D., Zheng G. (2020). Discovery of IAP-recruiting BCL-X(L) PROTACs as potent degraders across multiple cancer cell lines. Eur. J. Med. Chem..

[116] Zhang X., Thummuri D., He Y., Liu X., Zhang P., Zhou D., Zheng G. (2019). Utilizing PROTAC technology to address the on-target platelet toxicity associated with inhibition of BCL-XL. Chem. Commun. (Camb).

[117] Burslem G.M., Song J., Chen X., Hines J., Crews C.M. (2018). Enhancing antiproliferative activity and selectivity of a FLT-3 inhibitor by proteolysis targeting chimera conversion. J. Am. Chem. Soc..

[118] Cromm P.M., Samarasinghe K.T.G., Hines J., Crews C.M. (2018). Addressing kinase-independent functions of Fak via PROTAC-mediated degradation. J. Am. Chem. Soc..

[119] Hall M.D., Handley M.D., Gottesman M.M. (2009). Is resistance useless? Multidrug resistance and collateral sensitivity. Trends Pharmacol. Sci..

[120] Davis I.D., Martin A.J., Stockler M.R., Begbie S., Chi K.N., Chowdhury S., Coskinas X., Frydenberg M., Hague W.E., Horvath L.G., Joshua A.M., Lawrence N.J., Marx G., McCaffrey J., McDermott R. (2019). Enzalutamide with standard first-line therapy in metastatic prostate cancer. N. Engl. J. Med..

[121] Beltran H., Yelensky R., Frampton G.M., Park K., Downing S.R., MacDonald T.Y., Jarosz M., Lipson D., Tagawa S.T., Nanus D.M., Stephens P.J., Mosquera J.M., Cronin M.T., Rubin M.A. (2013). Targeted next-generation sequencing of advanced prostate cancer identifies potential therapeutic targets and disease heterogeneity. Eur. Urol..

[122] Grasso C.S., Wu Y.M., Robinson D.R., Cao X., Dhanasekaran S.M., Khan A.P., Quist M.J., Jing X., Lonigro R.J., Brenner J.C., Asangani I.A., Ateeq B., Chun S.Y., Siddiqui J., Sam L. (2012). The mutational landscape of lethal castration-resistant prostate cancer. Nature.

[123] Mayor-Ruiz C., Jaeger M.G., Bauer S., Brand M., Sin C., Hanzl A., Mueller A.C., Menche J., Winter G.E. (2019). Plasticity of the Cullin-RING ligase repertoire shapes sensitivity to ligand-induced protein degradation. Mol. Cell.

[124] Mogollón P., Díaz-Tejedor A., Algarín E.M., Paíno T., Garayoa M., Ocio E.M. (2019). Biological background of resistance to current standards of care in multiple myeloma. Cells.

[125] Ottis P., Palladino C., Thienger P., Britschgi A., Heichinger C., Berrera M., Julien-Laferriere A., Roudnicky F., Kam-Thong T., Bischoff J.R., Martoglio B., Pettazzoni P. (2019). Cellular resistance mechanisms to targeted protein degradation converge toward Impairment of the engaged ubiquitin transfer pathway. ACS Chem. Biol..

[126] Pfaff P., Samarasinghe K.T.G., Crews C.M., Carreira E.M. (2019). Reversible spatiotemporal control of induced protein degradation by bistable PhotoPROTACs. ACS Cent. Sci..

[127] Reynders M., Matsuura B.S., Bérouti M., Simoneschi D., Marzio A., Pagano M., Trauner D. (2020). PHOTACs enable optical control of protein degradation. Sci. Adv..

[128] Liu J., Chen H., Ma L., He Z., Wang D., Liu Y., Lin Q., Zhang T., Gray N., Kaniskan H., Jin J., Wei W. (2020). Light-induced control of protein destruction by opto-PROTAC. Sci. Adv..

[129] Ito T., Tanabe K., Yamada H., Hatta H., Nishimoto S.-i. (2008). Radiation- and photo-induced activation of 5-fluorouracil prodrugs as a strategy for the selective treatment of solid tumors. Molecules.

[130] Clift D., McEwan W.A., Labzin L.I., Konieczny V., Mogessie B., James L.C., Schuh M. (2017). A method for the acute and rapid degradation of endogenous proteins. Cell.

[131] Ibrahim A.F.M., Shen L., Tatham M.H., Dickerson D., Prescott A.R., Abidi N., Xirodimas D.P., Hay R.T. (2020). Antibody RING-mediated destruction of endogenous proteins. Mol. Cell.

[132] Tan X., Calderon-Villalobos L.I.A., Sharon M., Zheng C., Robinson C.V., Estelle M., Zheng N. (2007). Mechanism of auxin perception by the TIR1 ubiquitin ligase. Nature.

[133] Yesbolatova A., Saito Y., Kitamoto N., Makino-Itou H., Ajima R., Nakano R., Nakaoka H., Fukui K., Gamo K., Tominari Y., Takeuchi H., Saga Y., Hayashi K.-i., Kanemaki M.T. (2020). The auxin-inducible degron 2 technology provides sharp degradation control in yeast, mammalian cells, and mice. Nat. Commun..

[134] Kim J.H., Scialli A.R. (2011). Thalidomide: The tragedy of birth defects and the effective treatment of disease. Toxicol. Sci..

[135] Bartlett J.B., Dredge K., Dalgleish A.G. (2004). The evolution of thalidomide and its IMiD derivatives as anticancer agents. Nat. Rev. Cancer.

[136] Rehman W., Arfons L.M., Lazarus H.M. (2011). The rise, fall and subsequent triumph of thalidomide: Lessons learned in drug development. Ther. Adv. Hematol..

[137] Chanan-Khan A.A., Swaika A., Paulus A., Kumar S.K., Mikhael J.R., Rajkumar S.V., Dispenzieri A., Lacy M.Q. (2013). Pomalidomide: The new immunomodulatory agent for the treatment of multiple myeloma. Blood Cancer J..

[138] Kotla V., Goel S., Nischal S., Heuck C., Vivek K., Das B., Verma A. (2009). Mechanism of action of lenalidomide in hematological malignancies. J. Hematol. Oncol..

[139] Ito T., Handa H. (2019). [Cereblon as a primary target of IMiDs]. Rinsho Ketsueki.

[140] Fischer E.S., Böhm K., Lydeard J.R., Yang H., Stadler M.B., Cavadini S., Nagel J., Serluca F., Acker V., Lingaraju G.M., Tichkule R.B., Schebesta M., Forrester W.C., Schirle M., Hassiepen U. (2014). Structure of the DDB1–CRBN E3 ubiquitin ligase in complex with thalidomide. Nature.

[141] Gandhi A.K., Kang J., Havens C.G., Conklin T., Ning Y., Wu L., Ito T., Ando H., Waldman M.F., Thakurta A., Klippel A., Handa H., Daniel T.O., Schafer P.H., Chopra R. (2014). Immunomodulatory agents lenalidomide and pomalidomide co-stimulate T cells by inducing degradation of T cell repressors Ikaros and Aiolos via modulation of the E3 ubiquitin ligase complex CRL4(CRBN.). Br. J. Haematol..

[142] Krönke J., Udeshi N.D., Narla A., Grauman P., Hurst S.N., McConkey M., Svinkina T., Heckl D., Comer E., Li X., Ciarlo C., Hartman E., Munshi N., Schenone M., Schreiber S.L. (2014). Lenalidomide causes selective degradation of IKZF1 and IKZF3 in multiple myeloma cells. Science.

[143] Matyskiela M.E., Couto S., Zheng X., Lu G., Hui J., Stamp K., Drew C., Ren Y., Wang M., Carpenter A., Lee C.-W., Clayton T., Fang W., Lu C.-C., Riley M. (2018). SALL4 mediates teratogenicity as a thalidomide-dependent cereblon substrate. Nat. Chem. Biol..

[144] Petzold G., Fischer E.S., Thomä N.H. (2016). Structural basis of lenalidomide-induced CK1α degradation by the CRL4(CRBN) ubiquitin ligase. Nature.

[145] Sievers Q.L., Petzold G., Bunker R.D., Renneville A., Słabicki M., Liddicoat B.J., Abdulrahman W., Mikkelsen T., Ebert B.L., Thomä N.H. (2018). Defining the human C2H2 zinc finger degrome targeted by thalidomide analogs through CRBN. Science.

[146] Han T., Goralski M., Gaskill N., Capota E., Kim J., Ting T.C., Xie Y., Williams N.S., Nijhawan D. (2017). Anticancer sulfonamides target splicing by inducing RBM39 degradation via recruitment to DCAF15. Science.

[147] Bussiere D.E., Xie L., Srinivas H., Shu W., Burke A., Be C., Zhao J., Godbole A., King D., Karki R.G., Hornak V., Xu F., Cobb J., Carte N., Frank A.O. (2020). Structural basis of indisulam-mediated RBM39 recruitment to DCAF15 E3 ligase complex. Nat. Chem. Biol..

[148] Słabicki M., Kozicka Z., Petzold G., Li Y.-D., Manojkumar M., Bunker R.D., Donovan K.A., Sievers Q.L., Koeppel J., Suchyta D., Sperling A.S., Fink E.C., Gasser J.A., Wang L.R., Corsello S.M. (2020). The CDK inhibitor CR8 acts as a molecular glue degrader that depletes cyclin K. Nature.

[149] Mayor-Ruiz C., Bauer S., Brand M., Kozicka Z., Siklos M., Imrichova H., Kaltheuner I.H., Hahn E., Seiler K., Koren A., Petzold G., Fellner M., Bock C., Müller A.C., Zuber J. (2020). Rational discovery of molecular glue degraders via scalable chemical profiling. Nat. Chem. Biol..

[150] Lv L., Chen P., Cao L., Li Y., Zeng Z., Cui Y., Wu Q., Li J., Wang J.-H., Dong M.-Q., Qi X., Han T. (2020). Discovery of a molecular glue promoting CDK12-DDB1 interaction to trigger cyclin K degradation. Elife.

[151] Lipinski C.A., Lombardo F., Dominy B.W., Feeney P.J. (2001). Experimental and computational approaches to estimate solubility and permeability in drug discovery and development settings. Adv. Drug Deliv. Rev..

[152] Uhlén M., Fagerberg L., Hallström B.M., Lindskog C., Oksvold P., Mardinoglu A., Sivertsson Å., Kampf C., Sjöstedt E., Asplund A., Olsson I., Edlund K., Lundberg E., Navani S., Szigyarto C.A.-K. (2015). Tissue-based map of the human proteome. Science.

[153] Odenthal J., Takes R., Friedl P. (2016). Plasticity of tumor cell invasion: Governance by growth factors and cytokines. Carcinogenesis.

[154] Banik S.M., Pedram K., Wisnovsky S., Ahn G., Riley N.M., Bertozzi C.R. (2020). Lysosome-targeting chimaeras for degradation of extracellular proteins. Nature.

[155] Gary-Bobo M., Nirdé P., Jeanjean A., Morère A., Garcia M. (2007). Mannose 6-phosphate receptor targeting and its applications in human diseases. Curr. Med. Chem..

[156] Coutinho M.F., Prata M.J., Alves S. (2012). Mannose-6-phosphate pathway: A review on its role in lysosomal function and dysfunction. Mol. Genet. Metab..

[157] David C., Mengwen Z., Jason R., Jake S., Emily B., Egor C., Venkata S., Angela G., David M., Viswanathan M., David S. (2020). Bifunctional small molecules that mediate the degradation of extracellular proteins. ChemRxiv.

[158] Tanowitz M., Hettrick L., Revenko A., Kinberger G.A., Prakash T.P., Seth P.P. (2017). Asialoglycoprotein receptor 1 mediates productive uptake of N-acetylgalactosamine-conjugated and unconjugated phosphorothioate antisense oligonucleotides into liver hepatocytes. Nucleic Acids Res..

[159] Zhou Y., Teng P., Montgomery N.T., Li X., Tang W. (2021). Development of triantennary N-acetylgalactosamine conjugates as degraders for extracellular proteins. ACS Cent. Sci..

[160] Ahn G., Banik S.M., Miller C.L., Riley N.M., Cochran J.R., Bertozzi C.R. (2021). LYTACs that engage the asialoglycoprotein receptor for targeted protein degradation. Nat. Chem. Biol..

[161] Khandia R., Dadar M., Munjal A., Dhama K., Karthik K., Tiwari R., Yatoo M.I., Iqbal H.M.N., Singh K.P., Joshi S.K., Chaicumpa W. (2019). A comprehensive review of autophagy and its various roles in infectious, non-infectious, and lifestyle diseases: Current knowledge and prospects for disease prevention, novel drug design, and therapy. Cells.

[162] Nedelsky N.B., Todd P.K., Taylor J.P. (2008). Autophagy and the ubiquitin-proteasome system: Collaborators in neuroprotection. Biochim. Biophys. Acta Mol. Basis Dis..

[163] Mercer T.J., Gubas A., Tooze S.A. (2018). A molecular perspective of mammalian autophagosome biogenesis. J. Biol. Chem..

[164] Mari M., Griffith J., Rieter E., Krishnappa L., Klionsky D.J., Reggiori F. (2010). An Atg9-containing compartment that functions in the early steps of autophagosome biogenesis. J. Cell Biol..

[165] Herb M., Gluschko A., Schramm M. (2020). LC3-associated phagocytosis - the highway to hell for phagocytosed microbes. Semin. Cell Dev. Biol..

[166] Glick D., Barth S., Macleod K.F. (2010). Autophagy: Cellular and molecular mechanisms. J. Pathol..

[167] Anding A.L., Baehrecke E.H. (2017). Cleaning house: Selective autophagy of organelles. Dev. Cell.

[168] Sawa T., Zaki M.H., Okamoto T., Akuta T., Tokutomi Y., Kim-Mitsuyama S., Ihara H., Kobayashi A., Yamamoto M., Fujii S., Arimoto H., Akaike T. (2007). Protein S-guanylation by the biological signal 8-nitroguanosine 3′,5′-cyclic monophosphate. Nat. Chem. Biol..

[169] Sharma V., Verma S., Seranova E., Sarkar S., Kumar D. (2018). Selective autophagy and xenophagy in infection and disease. Front. Cell Dev. Biol..

[170] Ito C., Saito Y., Nozawa T., Fujii S., Sawa T., Inoue H., Matsunaga T., Khan S., Akashi S., Hashimoto R., Aikawa C., Takahashi E., Sagara H., Komatsu M., Tanaka K. (2013). Endogenous nitrated nucleotide is a key mediator of autophagy and innate defense against bacteria. Mol. Cell.

[171] Rawet-Slobodkin M., Elazar Z. (2013). 8-Nitro-cGMP—a new player in antibacterial autophagy. Mol. Cell.

[172] Takahashi D., Moriyama J., Nakamura T., Miki E., Takahashi E., Sato A., Akaike T., Itto-Nakama K., Arimoto H. (2019). AUTACs: Cargo-Specific degraders using selective autophagy. Mol. Cell.

[173] Li Z., Zhu C., Ding Y., Fei Y., Lu B. (2020). ATTEC: A potential new approach to target proteinopathies. Autophagy.

[174] Li Z., Wang C., Wang Z., Zhu C., Li J., Sha T., Ma L., Gao C., Yang Y., Sun Y., Wang J., Sun X., Lu C., Difiglia M., Mei Y. (2019). Allele-selective lowering of mutant HTT protein by HTT–LC3 linker compounds. Nature.

[175] Kanner S.A., Shuja Z., Choudhury P., Jain A., Colecraft H.M. (2020). Targeted deubiquitination rescues distinct trafficking-deficient ion channelopathies. Nat. Methods.

[176] Siriwardena S.U., Munkanatta Godage D.N.P., Shoba V.M., Lai S., Shi M., Wu P., Chaudhary S.K., Schreiber S.L., Choudhary A. (2020). Phosphorylation-inducing chimeric small molecules. J. Am. Chem. Soc..

[177] Conway S.J. (2020). Bifunctional molecules beyond PROTACs. J. Med. Chem..

[178] Yamazoe S., Tom J., Fu Y., Wu W., Zeng L., Sun C., Liu Q., Lin J., Lin K., Fairbrother W.J., Staben S.T. (2020). Heterobifunctional molecules induce dephosphorylation of kinases–A proof of concept study. J. Med. Chem..

